# The 4E-BP Caf20p Mediates Both eIF4E-Dependent and Independent Repression of Translation

**DOI:** 10.1371/journal.pgen.1005233

**Published:** 2015-05-14

**Authors:** Lydia M. Castelli, David Talavera, Christopher J. Kershaw, Sarah S. Mohammad-Qureshi, Joseph L. Costello, William Rowe, Paul F. G. Sims, Christopher M. Grant, Simon J. Hubbard, Mark P. Ashe, Graham D. Pavitt

**Affiliations:** 1 The Faculty of Life Sciences, The University of Manchester, Manchester, United Kingdom; 2 Manchester Institute of Biotechnology (MIB), The University of Manchester, Manchester, United Kingdom; The University of North Carolina at Chapel Hill, UNITED STATES

## Abstract

Translation initiation factor eIF4E mediates mRNA selection for protein synthesis via the mRNA 5’cap. A family of binding proteins, termed the 4E-BPs, interact with eIF4E to hinder ribosome recruitment. Mechanisms underlying mRNA specificity for 4E-BP control remain poorly understood. Saccharomyces cerevisiae 4E-BPs, Caf20p and Eap1p, each regulate an overlapping set of mRNAs. We undertook global approaches to identify protein and RNA partners of both 4E-BPs by immunoprecipitation of tagged proteins combined with mass spectrometry or next-generation sequencing. Unexpectedly, mass spectrometry indicated that the 4E-BPs associate with many ribosomal proteins. 80S ribosome and polysome association was independently confirmed and was not dependent upon interaction with eIF4E, as mutated forms of both Caf20p and Eap1p with disrupted eIF4E-binding motifs retain ribosome interaction. Whole-cell proteomics revealed Caf20p mutations cause both up and down-regulation of proteins and that many changes were independent of the 4E-binding motif. Investigations into Caf20p mRNA targets by immunoprecipitation followed by RNA sequencing revealed a strong association between Caf20p and mRNAs involved in transcription and cell cycle processes, consistent with observed cell cycle phenotypes of mutant strains. A core set of over 500 Caf20p-interacting mRNAs comprised of both eIF4E-dependent (75%) and eIF4E-independent targets (25%), which differ in sequence attributes. eIF4E-independent mRNAs share a 3’ UTR motif. Caf20p binds all tested motif-containing 3’ UTRs. Caf20p and the 3’UTR combine to influence *ERS1* mRNA polysome association consistent with Caf20p contributing to translational control. Finally *ERS1* 3’UTR confers Caf20-dependent repression of expression to a heterologous reporter gene. Taken together, these data reveal conserved features of eIF4E-dependent Caf20p mRNA targets and uncover a novel eIF4E-independent mode of Caf20p binding to mRNAs that extends the regulatory role of Caf20p in the mRNA-specific repression of protein synthesis beyond its interaction with eIF4E.

## Author Summary

In eukaryotic cells protein synthesis initiation factor eIF4E controls mRNA selection by interacting with the mRNA 5’ cap. A family of binding proteins, termed the 4E-BPs, interact with eIF4E to hinder ribosome recruitment and repress translation of their target mRNAs. The yeast *Saccharomyces cerevisiae* has two 4E-BPs Caf20p and Eap1p that regulate distinct but overlapping sets of mRNAs. Here, we describe genome wide experiments to identify protein and RNA partners of each 4E-BP, with a greater focus on Caf20p. We present evidence that the 4E-BPs unexpectedly bind to ribosomes, an interaction that is not dependent on eIF4E binding. We also define a core set of over 500 Caf20p target mRNAs that fall into two classes with distinct features. One mRNA class, representing 25% of the targets, binds Caf20p independently of its eIF4E interaction and instead via a novel 3’ UTR interaction. Our data indicate that these proteins can repress mRNA-specific protein synthesis independently of their known role as eIF4E-binding proteins.

## Introduction

Translation is a dynamic and multi-step process involving a multitude of interactions between the ribosome, RNAs and protein factors to produce the complement of proteins required for life. Operationally it is divided into distinct initiation, elongation and termination phases; each requiring distinct sets of protein synthesis factors. Control of the translation of a large number of mRNAs has been shown to occur at the rate-limiting initiation phase, thereby allowing rapid cellular responses to a wide variety of stimuli [1]. Translation initiation involves at least 12 proteins, which act in concert to form a series of ribonucleoprotein complexes that result in an 80S ribosomal complex primed with initiator tRNA and bound precisely at the mRNA start codon, ready to begin translation elongation [2].

Two major initiation steps targeted for control are (i) the GTP-dependent binding of initiator tRNA to eIF2, to form a ternary complex, which with other factors primes 40S ribosomes for protein synthesis initiation, and (ii) mRNA selection via the eIF4F 5’ mRNA cap-binding complex comprised of eIF4E, eIF4G and the polyA tail binding protein Pab1p [1,3]. The first of these control mechanisms is through the activation of eIF2α kinases [4]. Phosphorylation of the alpha subunit of eIF2 results in a block of the activity of eIF2B and therefore impairs the recycling of eIF2•GDP to eIF2•GTP and ultimately reducing the amount of ternary complex available for translation initiation [5–7]. Phosphorylation of eIF2 occurs in all eukaryotes studied and in response to diverse regulatory cues. Although widely inhibitory to active protein synthesis, it also ensures activation of translation of specific mRNAs to mediate critically important translational control mechanisms including genes bearing upstream open reading frames including the classic examples ATF4 and *GCN4* [1,6].

A second major translation initiation regulatory mechanism involves disruption of the eIF4F complex that is important for mRNA selection. mRNAs possess a 5’ 7-methylguanosine cap to which eIF4E binds. eIF4E in turn interacts with eIF4G to form the eIF4F complex that promotes 40S ribosome recruitment. eIF4E-binding proteins (4E-BPs) can compete with eIF4G for a shared binding site on the surface of eIF4E. The current accepted model is that association between a 4E-BP and eIF4E prohibits eIF4E-eIF4G interaction thereby causing repression of translation [8]. 4E-BPs and eIF4G share a degenerate motif (YXXXXLϕ, where ϕ is Leu, Met or Phe and X is any amino acid) that is important for their interaction with eIF4E. 4E-BPs can inhibit translation on a global scale through a general down regulation of 5’ cap-dependent translation initiation or via more mRNA specific means. For example, in mammalian cells mTOR promotes cell growth and phosphorylates and inactivates 4E-BP1. However inhibiting mTOR either chemically or during nutrient starvation leads to reduced phosphorylation of 4E-BP1. This in turn activates 4E-BP1/eIF4E binding, repressing translation [8,9]. mRNA-specific inhibition of translation is demonstrated by other 4E-BPs with selectivity coupled to interactions between the 4E-BP and mRNA-specific binding proteins. For example Cup in *Drosophila melanogaster* is a 4E-BP that interacts with the RNA-binding protein Bruno to repress translation of *oskar* mRNA [10] and with Smaug to repress *nanos* mRNA translation [11].

*Saccharomyces cerevisiae* has two 4E-BPs: Caf20p (also known as p20) [12] and Eap1p [13]. In common with their metazoan homologues, both compete with eIF4G for binding to eIF4E via the shared conserved interaction motif [14], suggesting that they provide a good model system to compare with their metazoan counterparts. Each gene is non-essential for growth and several studies have suggested roles for each gene including the developmental induction of pseudohyphae in response to nitrogen limitation [14]. More recent studies have indicated a variety of roles especially for Eap1p. It is required for translational control in response to certain oxidative stress agents [15] and is associated with stress granules during glucose starvation [16]. In addition, Eap1p may play roles in mRNA decay, as it has been associated with promoting mRNA decapping [17, 18].

Previously we performed transcriptome-wide analyses to assess the impact of loss of each 4E-BP gene, individually, on the engagement of mRNAs with ribosomes in actively growing cells [19]. Our study revealed that the association of over 1000 mRNAs with polyribosomes was modulated by the deletion of *EAP1* or *CAF20*, suggesting each 4E-BP was able to bind and regulate translation of a large fraction of mRNAs. In a follow-up study, we recently used affinity purification and high throughput RNA sequencing to identify RNAs preferentially associated with eIF4E, each eIF4G isoform, the polyA binding protein and each 4E-BP [20]. This approach showed that each 4E-BP shares a large overlap in its target RNAs, with over 1000 mRNAs enriched by each factor. Conversely many mRNAs are not enriched with either factor [20]. These data reinforce the idea that there is specificity to the mRNAs with which each 4E-BP interacts, but did not illuminate the mechanisms involved. Curiously the mRNAs enriched by the 4E-BPs were not all enriched with eIF4E suggesting that the 4E-BPs may have additional partners [20].

In this study we set out to identify protein-binding partners for Caf20p and Eap1p that may mediate mRNA specificity using mass spectrometry. We find that in addition to their known binding partner eIF4E, both factors interact with intact ribosomes and polysomes. Ribosome and eIF4E binding are independent as 4E-BPs mutated to disrupt eIF4E binding retain ribosome interaction and some other functions. Similarly RNA-sequencing of Caf20p-bound mRNAs identified a core set of over 500 bound mRNAs, 25% of which are independent of the Caf20p-eIF4E interaction. Caf20p-binding represses translation of its targets and we identify distinct features of eIF4E-dependent and eIF4E-independent Caf20p-binding RNAs. Taken together our data reveal sequence features of eIF4E-dependent Caf20p transcripts and show that in addition to their role of repressing translation via eIF4E, the 4E-BPs can repress translation via a novel mechanism that is physiologically relevant and that likely involves both ribosome interaction and 3’ UTR binding, possibly via a conserved sequence element within the 3’UTRs of mRNA targets.

## Results

### 4E-BPs associate with other RNA binding proteins

**Fig 1:**
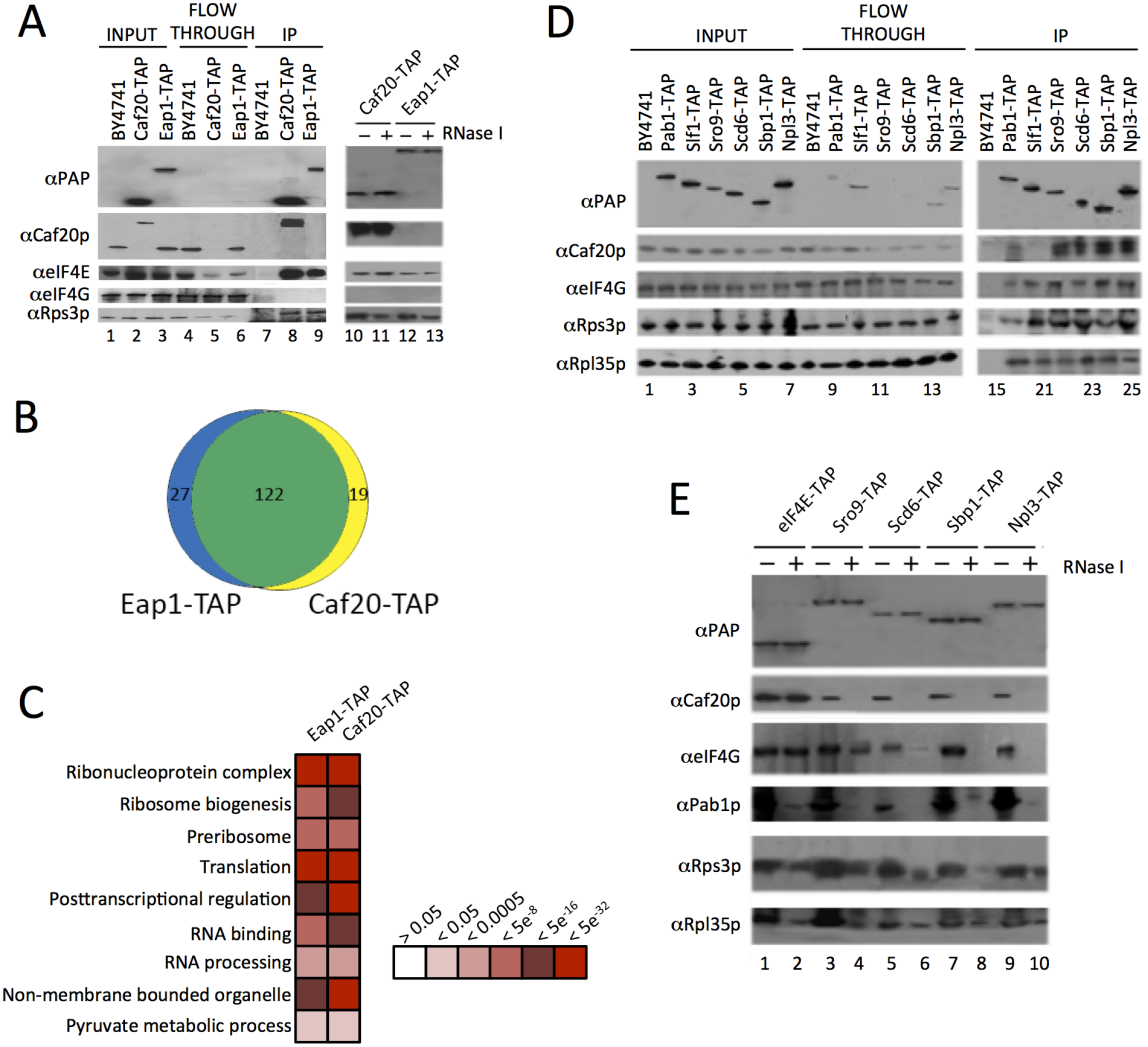
Identification of 4E-BP associating proteins using TAP-tag IPs. A. Western blots of the TAP IPs, lanes 1–9 show input, flowthrough and eluates for the IP strains and lanes 10–13 show the eluates of the 4E-BPs with and without RNase I digestion. Blots were probed with the α Protein A peroxidase (PAP) antibody (detects the TAP-tag), αCaf20p, αeIF4E, αeIF4G and αRps3p to highlight IP efficiency and maintenance of previously published associations. B. Proportional Venn-style diagram depicting the overlaps between proteins associating with the 4E-BPs. C. Gene Ontology classes statistically overrepresented within 4E-BP associated proteins at indicated FDR significance. Only a representative selection of the significant functional categories is shown D. Western blots from reciprocal TAP IPs of a selection of RNA-binding proteins identified as interacting with the 4E-BPs probed with the indicated antibodies. E. Effect of RNase I digestion on protein interactions identified in D.

Microarray analysis comparing the polysome association of mRNAs in wild type cells with those deleted singly for each yeast 4E-BP has shown that the two proteins, Caf20p and Eap1p, regulate the polysome association of over 1000 yeast mRNAs [19]. The experiments indicate that there are both overlapping and distinct mRNAs regulated by each factor, implying that there is some mRNA specificity underlying their regulation of translation. These ideas were reinforced by a recent follow-up study in which we used affinity purification and high throughput RNA sequencing to identify RNAs preferentially associated with eIF4E, each eIF4G isoform, the polyA binding protein and each 4E-BP [20]. To assess the mechanisms by which the yeast 4E-BPs are targeted to different mRNAs, the protein interactions of Caf20p and Eap1p were investigated here using tandem affinity purification (TAP) tagged factors and mass spectrometry. Unlike prior large-scale studies [21,22], we employed magnetic beads coated with IgG that have reduced non-specific interactions and that permit rapid isolation of the protein A moiety of the TAP tag [23], combined with a competitive peptide elution step to recover bound proteins [24]. We first confirmed by western blotting that Caf20-TAP and Eap1-TAP both co-immunoprecipitate eIF4E, but not eIF4G (Fig 1A) as previously described [14,19]. No association between the two 4E-BPs was observed, as expected (Fig 1A). Five replicates of each TAP purification were trypsin digested and processed by label-free LC-MS/MS followed by identification and relative quantification of bound peptide abundances. This analysis revealed that Caf20p associated with 116 proteins (FDR<0.05, 2 or more peptides used for quantification) and Eap1p bound 118 proteins. The large overlap (101 proteins) between both experiments suggests there is little to distinguish between the 4E-BP protein interactions (Fig 1B). Functional classification of the associating proteins, using the Database For Annotation, Visualization And Integrated Discovery (DAVID), highlighted enrichment of various categories (Fig 1C). Over half the proteins significantly enriched were ribosomal proteins (80, including 69 identified with both TAP proteins) with no clear bias toward 40S or 60S. An additional 20 proteins found have roles in ribosome biogenesis and further ribosome-associated proteins were also found (S1 Dataset, sheet 1 and 2). Although ribosomal proteins are frequent contaminants of this type of analysis, the large number of proteins identified and quantified warranted further analysis. This is described in the following section. In addition we found proteins involved in RNA processing and RNA binding including components of the mRNA decay pathway such as Xrn1p, Nam7p and Dbp2. Roles for both Caf20p and Eap1p in mRNA decay have been suggested previously [17,18,25,26].

Associations between the yeast 4E-BPs and RNA binding proteins have been postulated to modulate 4E-BP mRNA specificity [19]. To validate new potential RNA-binding partner candidates, reciprocal TAP immunoprecipitations (IPs) were performed for a selection of RNA binding proteins (Fig 1D). The RNA binding proteins selected included proteins identified by MS and some related proteins which were not found and which acted as controls. The La related protein Sro9p was found associated with both Caf20-TAP and Eap1-TAP, while its homolog Slf1p was not and was included as a negative control. Both Slf1p and Sro9p associate with actively translating ribosomes, or polysomes [27]. Caf20p was found to co-IP with Sro9-TAP, but not Slf1-TAP (Fig 1D). This data suggests that Caf20p is not interacting with all ribosome associated factors and agrees with a separate study examining the role of Slf1p [28]. The remaining RNA binding proteins we analysed here included the polyA binding protein Pab1p, as a positive control, and three proteins characterised as eIF4G associating translational repressors [29]: Npl3p and Sbp1p were associated with both 4E-BP-TAP IPs, and Scd6p which was not identified here. All IPs were probed for association with Caf20p, eIF4G and the ribosomal protein markers to validate previous observations. No antibody is available for Eap1p and our attempts to generate one proved unsuccessful, so only Caf20p interactions were examined. Caf20p was found to co-immunoprecipitate with all the TAP tagged proteins chosen, with the expected exception of Slf1-TAP (Fig 1D). Scd6p associated with Caf20p, despite not being detected by our MS analyses, highlighting that MS did not provide a complete set of associating proteins.

Association between known eIF4G binding proteins and Caf20p (Fig 1D), where no direct binding between Caf20p and eIF4G was expected or found (Fig 1A), suggested that some of the protein interactions identified are likely to be RNA-dependent. To assess RNA-dependence, IPs were repeated on samples treated with RNase I (Fig 1A and 1E). This showed that Caf20p and Eap1p associate with eIF4E directly (Fig 1A), but that binding between Caf20p and all the other RNA-binding proteins tested was RNase sensitive (Fig 1E). We also probed the IPs with ribosomal proteins (Rps3p and Rpl35p), which confirmed that ribosomes associated in all the TAP IPs (between 1–3% of the total in input samples) and that ribosome interactions were diminished by RNase I treatment. These data suggested that the interactions we were observing were largely as a consequence of the interaction between the 4E-BPs and ribosomes, or ribosome-associated RNAs. As 4E-BPs are generally thought to be repressors of translation, we decided to examine 4E-BP and ribosome binding more closely.

### 4E-BPs bind translating ribosomes

**Fig 2:**
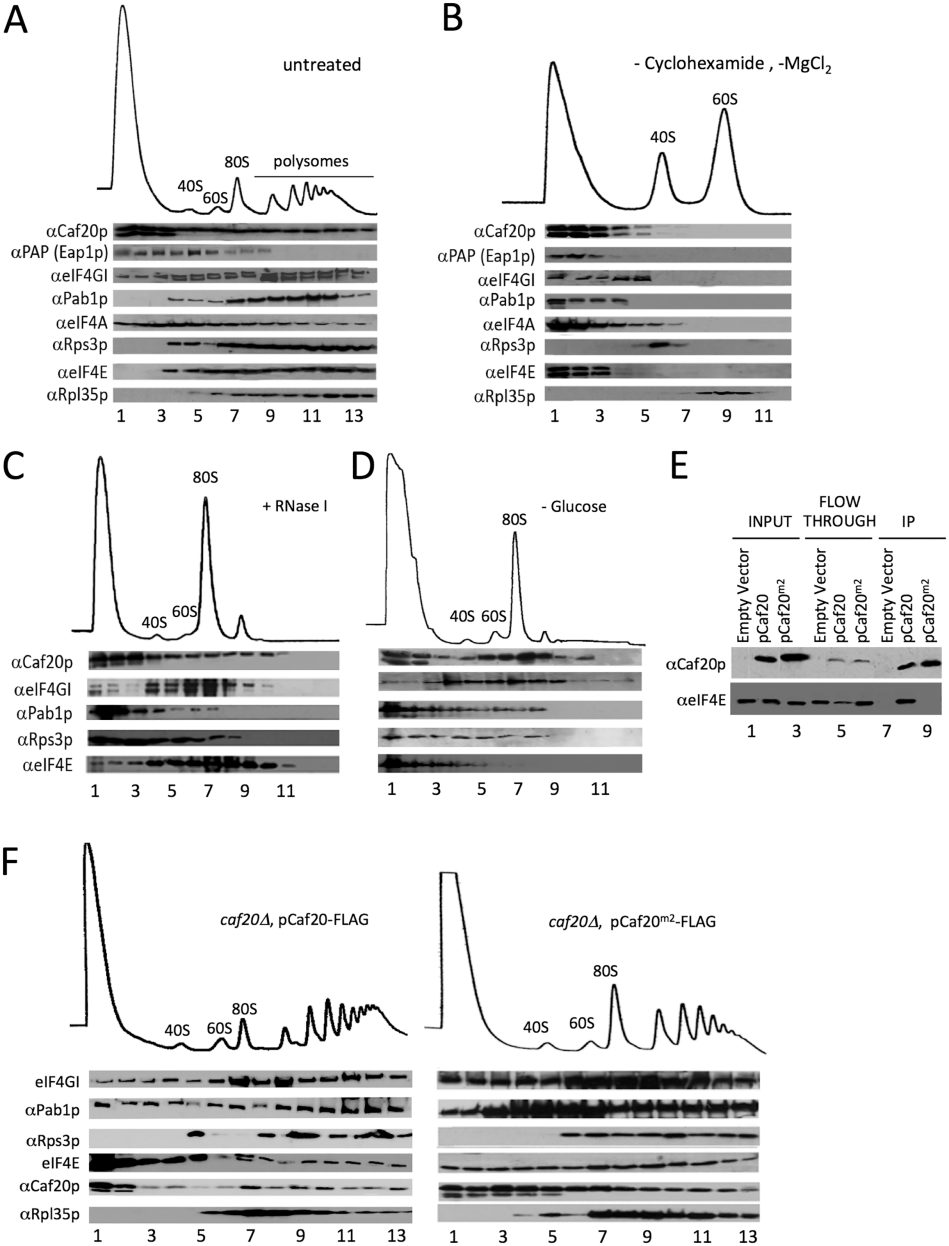
4E-BPs associate with translating ribosomes in an eIF4E-independent manner. A. Sucrose density gradient analysis of extracts from the Eap1-TAP strain. Fractions were collected and protein distribution across the gradients visualised with western blots. Blots were probed for the 4E-BPs, closed loop components and 40S/60S ribosomal subunits. B. Western blot analyses of fractions collected from sucrose density gradients of extracts from the Eap1-TAP strain. The 80S ribosomal complex was dissociated by removal of cycloheximide and MgCl_2_ from the lysis buffer. Blots were probed for the 4E-BPs, closed loop components and 40S/60S ribosomal subunits. C. Western blot analysis of fractions collected from a sucrose density gradient of extract from the BY4741 *HIS3* strain GP6001. The lysate was pre-treated with RNase I. D. Western blot analysis of fractions collected from a sucrose density gradient of extracts from the BY4741 *HIS3* strain which had been grown in SCD-HIS and resuspended in SC-HIS for 10 min prior to cycloheximide treatment. E. Western blot analysis of FLAG immune-purification of whole cell extracts from *caf20Δ* strains harbouring an empty vector [pRS426], p[*CAF20-FLAG*] or p[*CAF20* ^*m2*^-*FLAG*]. F. Western blot analysis of fractions collected from sucrose density gradients of extracts from the p[*CAF20-FLAG*] and p[*CAF20* ^*m2*^-*FLAG*] strains.

Our IPs and MS analyses identified ribosomal proteins including a wide representation of both small and large ribosomal subunit proteins (80 proteins in total). To validate this observation, the distribution of the 4E-BPs across polysome profiles was assessed for cell extracts treated with cycloheximide to ‘freeze’ ribosomes translating mRNAs. Western blotting of sucrose gradient fractions showed that Caf20p is distributed across both ribosome free and ribosome associated fractions in a manner similar to many translation initiation factors (Fig 2A). Eap1-TAP distribution differed as it was not enriched in polysomal fractions (lanes 10–13). Disrupting ribosomes by eliminating cycloheximide and magnesium from buffers leads to separation of 40S and 60S subunits. The 4E-BPs did not retain binding to either subunit under these conditions (Fig 2B). Similarly ribosomal association of the 4E-BPs, as assessed by sucrose cushion gradients, was disrupted by increased salt in the buffer (S1 Fig). Together these studies show that the 4E-BPs behave similar to the translation factors we probed for in these experiments, rather than as core ribosomal subunit proteins. RNase I digestion of cycloheximide treated cell extracts prior to separation eliminates polysomes, but preserves intact 80S complexes (Fig 2C). Similarly glucose starvation of cells for 10 min prior to cycloheximide addition causes severe inhibition of translation, so that ribosomes migrate almost entirely as free 80S complexes with almost no polysomes (Fig 2D). Under both treatment regimes a major fraction of Caf20p remains associated with the 80S peak observed. These experiments demonstrate clearly that Caf20p can bind 80S ribosomes and polyribosomes and complement experiments published recently showing Eap1p also binds translating ribosomes [18].

### Association of 4E-BPs with ribosomes is eIF4E independent

The 4E-BPs strongly associate with eIF4E (Fig 1A) through a conserved motif shared with eIF4G. A double alanine substitution Y4A, L9A (called Caf20^*m2*^p) has previously been shown to disrupt eIF4E-binding [14]. To investigate the role eIF4E-binding plays in targeting Caf20p to ribosomes and its other protein targets, we generated a *caf20*Δ strain complemented with a plasmid expressing C-terminally FLAG-Tagged wild type Caf20p or the Caf20^*m2*^p mutations. Caf20p and Caf20^*m2*^p were captured from extracts of these cells on magnetic anti-FLAG beads with equivalent efficiencies. However as expected, only wild type Caf20-FLAG bound to eIF4E (Fig 2E). This experiment confirms that Caf20^*m2*^p mutations disrupt its binding to eIF4E in our cells. Despite this, Caf20^*m2*^-FLAG retained its ability to associate with translating ribosomes (Fig 2F), suggesting that Caf20p ribosome association is independent of eIF4E binding. Sedimentation through sucrose cushions that partition cell extracts into ribosome free (supernatant) and ribosome bound (pellet) fractions was used as an additional test of ribosome binding. Caf20^*m2*^p behaved identically to Caf20p in this assay, demonstrating binding to ribosomes that was disrupted by increasing salt levels (S1A and S1B Fig). We also examined strains bearing haemagglutinin epitope-tagged HA-Eap1p or its equivalent eIF4E-binding mutant (called HA-Eap1p^*m3*^) in the same sedimentation assay. Both proteins bound ribosomes and lost their ribosomal association with increasing salt concentrations (S1C and S1D Fig). Taken together these experiments demonstrate that ribosomal association of a significant fraction of the 4E-BPs is not dependent on its known interaction with eIF4E.

### Altered proteome profiles of 4E-BPΔ strains provide evidence for both eIF4E dependent and independent roles

To examine the impact of loss or mutation of the 4E-BPs on the steady-state proteome, we performed whole cell proteomic analyses on whole-cell lysates from wild type, *caf20Δ*, *eap1Δ*, *caf20Δeap1Δ* double deleted strains. In addition, to assess the dependence of caf20p changes observed on the eIF4E-binding motif we analysed our Caf20^*m2*^-FLAG strain. Following LC-MS/MS the identified peptides were analysed to determine relative changes in protein abundance between wild type and mutant strains. In total 1636 proteins were quantified across all samples (two or more peptides used for quantification). Comparing the proteins identified with their abundances (PaxDB data) indicates that, unsurprisingly, our datasets are biased towards abundant proteins (ie >70% of the proteins we identified are in the top 25% of all yeast protein abundances and almost none in the lower 50%). Both enrichment and depletion of proteins was detected amongst the different mutant strains when each was compared to the wild type (S1 Dataset, sheets 3–6). We detected changes in ~200–400 proteins (FDR<0.05) for each experiment.

**Fig 3:**
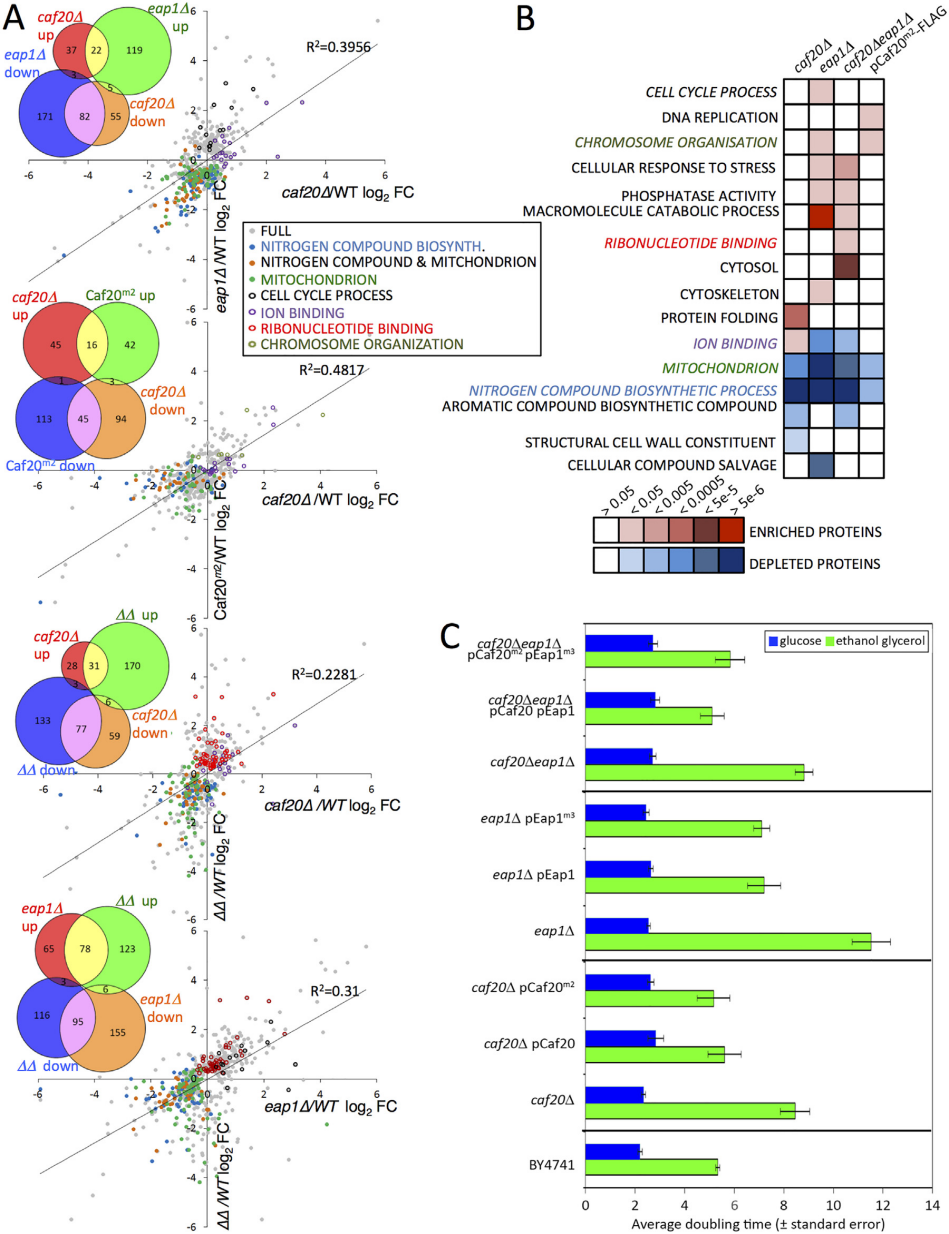
The altered proteome of single, double 4E-BPΔ and Caf20^*m2*^-FLAG strains. A. Pairwise comparisons of changes to proteome profiles for the 4E-BPΔ strains plotting log2 fold changes (log2FC) of each mutant strain to wild type (WT), showing only significantly changing proteins (FDR<0.05, 2 or more peptides used for quantification). Highlighted are key Gene Ontology (GO) classes that are over/under-represented. Colours are indicated in the inserted key. Lines of best fit and R^*2*^ correlation coefficients are shown. Four inset Venn-style diagrams in each upper left quadrant indicate numbers of proteins shared between datasets. ΔΔ indicates the double caf20Δ eap1Δ strain. B. Functional GO class analysis of the 4E-BPΔ and Caf20^*m2*^-FLAG altered proteomes using the DAVID Bioinformatics database. Over/underrepresentation colours depict FDR significance as per the key. Coloured text GO terms are highlighted in A. Only a representative selection of the significant functional categories is shown. C. Mean doubling times of the BY4741 (Wild type) and 4E-BPΔ strains bearing empty vector or plasmid copies of the indicated genes in fermentative (SCD) and respiratory (SCGE) media. Doubling times shown are an average of 3 biological replicates ± standard error.

Fig 3A shows pairwise comparisons between the experiments that demonstrate significant overlaps as well as distinct protein changes between each strain, illustrated using scatter plots and Venn style diagrams. Loss of Eap1p had greater impact than loss or mutation of Caf20p. Caf20p and Eap1p likely compensate for each other in the regulation of many mRNAs, as the *caf20Δeap1Δ* double deleted strain alters more proteins than either single deletion (Fig 3A). The plots also reveal that the *caf20*Δ and caf20^*m2*^p strains are most similar (R^2^ = 0.48), with many protein fold changes falling close to an approximate X = Y diagonal. The data suggest that these protein changes observed in the *caf20Δ* strain can be attributed to loss of eIF4E-binding. However other changes differ between these strains, which implies that regulating translation through eIF4E-binding is not the sole mode of action for Caf20p. As Caf20^*m2*^p retains its ribosome-binding function these data support the idea that Caf20p has both eIF4E dependent and independent roles in regulating protein abundance.

Functional analysis of the altered protein datasets revealed both similarities and distinct class enrichments amongst the different categories (Fig 3B). ‘Ion Binding’ was upregulated in *caf20Δ* cells, while ‘Cell Cycle’ was enriched in *eap1Δ* cells, and ‘Ribonucleotide Binding’ was enriched significantly in the double 4E-BPΔ strain. The most striking findings of our proteomics analyses are that ‘nitrogen compound metabolic process’ and ‘mitochondrial proteins’ were less abundant in each single 4E-BP deletion strain, the double mutant and Caf20^*m2*^p (Fig 3A and 3B). This may help explain growth defects observed when *caf20*Δ and *eap1*Δ strains were grown on a variety of amino acids as sole nitrogen sources [19]. To assess the importance of Caf20p and Eap1p and the eIF4E-binding motif in each factor for growth under conditions where mitochondria are critical, strain growth rates were monitored in glucose medium (fermentative growth) as well as in glycerol/ethanol medium (respiratory growth). As expected all cells grow more slowly using glycerol/ethanol, however each 4E-BP deletion strain grew very poorly in respiratory growth medium, especially *eap1*Δ (Fig 3C). The growth phenotypes were rescued when each deletion strain was complemented by a plasmid bearing the corresponding 4E-BP gene (Fig 3C). Interestingly the growth phenotypes were not dependent upon the eIF4E-4E-BP interaction motifs in each protein, as mutant variants of each 4E-BP also rescue the slow growth phenotype (Fig 3C). Taken together these data support the idea that each 4E-BP acts in part through its interaction with eIF4E, but that they have additional functionally important roles in regulating protein abundance that are independent of the eIF4E-binding motifs and eIF4E interactions.

### Defining a core set of Caf20p target mRNAs

**Fig 4:**
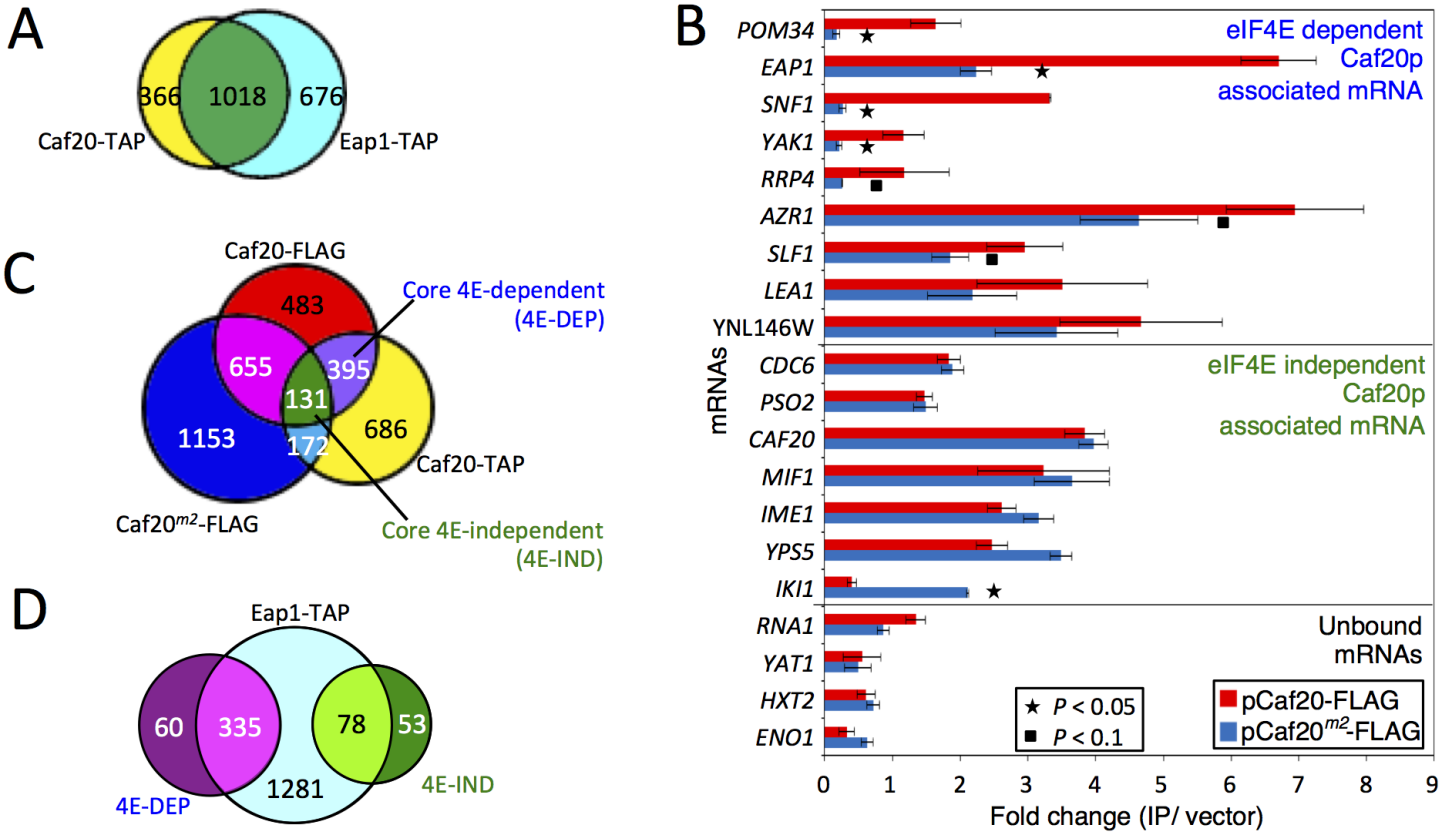
Identifying eIF4E-dependent and independent mRNAs associating with Caf20p. A. Proportional Venn style diagram showing the overlap between the mRNAs associating with Caf20p and Eap1p TAP IPs. B. Quantitative RT-PCR of selected mRNAs showing their association with Caf20p and Caf20^m2^p represented as fold-change over control. Statistically significant changes are indicated with a key (T-test). C. and D. Venn style diagrams showing C. mRNAs associating with Caf20-TAP, Caf20-FLAG and Caf20^*m2*^-FLAG, highlighting the core mRNAs identified in both studies and their dependence on the eIF4E interaction motif for binding, and D. Overlap between the core 4E-DEP and 4E-IND Caf20p interacting mRNAs and the Eap1-TAP associated proteins.

Previously we employed polysome separation of mRNAs and microarray analyses of *caf20*Δ and *eap1*Δ strains that showed regulation of a subset of approximately 1000 transcripts, many of which were altered by loss of both proteins [19]. It was not clear from the study which specific changes were caused directly by loss of 4E-BP binding and which were indirect consequences of 4E-BP loss. To extend this analysis we recently performed RNA sequencing of TAP-tagged protein captured mRNAs using the same IgG-bound magnetic bead-capture method that we used here for MS analysis. In that study we performed RNA-Seq experiments with Caf20-TAP, Eap1-TAP as well as eIF4E-TAP, eIF4G1-TAP, eIF4G2-TAP and PAB1-TAP [20]: factors that associate with mRNA 5’ caps or polyA tails. The analysis revealed 1694 RNAs bound to Eap1-TAP and 1384 to Caf20-TAP (FDR<0.05 significance cut-off), with a large overlap between the RNAs associating with each factor (Ref 20 and Fig 4A), implying significant redundancy in mRNA targets. Conclusions of the study include that yeast mRNAs fall into four broad classes based on which factors they are enriched with. Two classes of mRNA (termed groups II and IV) were enriched for either one or both 4E-BPs and both classes of mRNAs are associated with low ribosome occupancy levels. In contrast two classes were depleted for the 4E-BPs and are associated with higher ribosome occupancy. In addition, mRNAs enriched by the 4E-BPs were not all captured equally with eIF4E suggesting that the 4E-BPs may have additional partners [20].

As we have now found that Caf20p associates with proteins other than eIF4E (Figs 1 and 2, S1 Dataset) and maintains association with the ribosome even in the absence of the eIF4E-interaction motif, we wished to assess the eIF4E-dependence of Caf20p-associated mRNAs. We therefore performed quantitative RT-PCR analyses on a selection of mRNA targets using our FLAG-tagged *CAF20* and *caf20* ^*m2*^ strains following capture on anti-FLAG magnetic resin (Fig 4B). In addition to confirming that Caf20-TAP mRNA targets also bind Caf20-FLAG, this analysis showed that binding of many of the selected mRNAs to Caf20p is only partially diminished (eg *YNL146W*, *AZR1*, *LEA1*, *SLF1*) or largely/entirely independent of the eIF4E interaction motif, as they remain associated with Caf20^*m2*^-FLAG (eg *CAF20*, *IME1*, *MIF1* etc). To extend our analysis we performed new RNA-Seq experiments on RNAs captured by anti-FLAG magnetic resin from our *CAF20*-FLAG and *CAF20* ^*m2*^-FLAG strains. Each experiment was performed in triplicate and the full processed data are shown in the S1 Dataset (sheets 7 and 8). Because this RNA Sequencing experiment used a different tag and affinity-capture resin as well as a different sequencing platform (Illumina HiSeq here versus SOLiD 4), it provided a robust test of Caf20p-interacting RNAs. Caf20-FLAG captured 1664 transcripts (FDR <0.05), of which 526 were in common with our recent study (Fig 4C and S2 Fig). While statistically significant (*P* = 1.94e-16, cumulative hypergeometric distribution test), the overlap was less than might have been anticipated between this study and our prior TAP tagged data. One interpretation is that each experiment captures a fraction of all real interactions, implying that Caf20p binding to mRNAs is general rather than specific. Alternatively the statistical analysis may not reflect the actual false discovery rate. Plotting the mean fold changes for each experiment suggests that there is less variation (in terms of fold changes between captured mRNAs and total mRNA) than is suggested by those statistically enriched by both approaches (S2A Fig). However by applying the statistical threshold to both datasets we suggest that the 526 mRNAs identified by both independent approaches represent a ‘core’ set of Caf20p interacting mRNAs. Surprisingly Caf20^*m2*^-FLAG enriched 2111 mRNAs (using FDR<0.05), 958 of which (45%) were in common with either FLAG or TAP-tagged wild type Caf20, but of which only 131 appeared in our defined ‘core’ set of Caf20-interactors. Comparing the fold changes (enriched mRNAs/total mRNA) between wild type and mutant proteins, suggests that there is greater variation between these datasets than between the two wild type datasets (S2 Fig).

Of the defined 526 core Caf20p-bound mRNAs, 395 (75%) were dependent on the eIF4E-interaction motif and were not enriched in Caf20^*m2*^-FLAG IPs. These we operationally defined as core eIF4E-dependent mRNAs (4E-DEP; S1 Dataset, Sheet 9) for our subsequent bioinformatics analysis, described below. The remaining 131 (25%) of the core set were significantly associated with both Caf20-FLAG and Caf20^*m2*^-FLAG. These results both confirmed and extended our RT-PCR results that RNAs can bind Caf20^*m2*^p (Fig 4B), we therefore termed this subset of the core targets as eIF4E-independent (4E-IND; S1 Dataset, Sheet 10). A majority of the core set of Caf20p-interacting transcripts were also bound by Eap1-TAP, confirming the high degree of overlap between both 4E-BP mRNA targets (Fig 4D).

Our RNA sequencing experiment also revealed a further unexpected class of RNA. These are RNAs that only interact with mutated Caf20^*m2*^-FLAG (Fig 4C and S2B Fig). *IKI1*, encoding a factor required for tRNA modification, is one such mRNA, which was also seen by RT-PCR (Fig 4B). Over 1000 additional mRNAs were captured by Caf20^*m2*^p that were not enriched by the wild type protein, such that the number of Caf20^*m2*^p bound mRNAs was greater than for the wild type protein. These findings highlight that loss of eIF4E-binding is not neutral as the mutant protein apparently gains the ability to bind numerous new RNAs, possibly in part via enhanced ribosome interaction.

### Association with ribosome nascent chains does not explain eIF4E-independence

Because ribosome-associated protein nascent chains can fold into domains during protein synthesis, they may interact with protein binding partners as they are synthesized [30]. Hence a protein nascent chain binding to either 4E-BP may enrich for mRNAs indirectly. Such a mechanism would contribute to mRNA binding in our experiments and thereby explain some observed interactions. To assess if this is occurring at a significant level, we compared the identities of the proteins co-precipitating with Caf20-TAP in our TAP-IP MS analyses (Fig 1B) with our defined core Caf20p mRNA targets. Only two co-regulated mRNAs and protein pairs were found, suggesting co-translational interactions between 4E-BPs and mRNAs is not a general feature of these data. *TOP2* mRNA (encoding DNA topoisomerase 2) is among eIF4E-dependent core mRNAs (4E-DEP) and Top2p is associated with Eap1-TAP. The second mRNA is *CAF20* itself, with associates with Caf20p in a 4E-IND manner. This may indicate that Caf20p has an autoregulatory role, which we also suggested previously [20].

We also wished to assess overlap between our core Caf20p RNA targets and the changes we observed in our proteomics analyses following loss of either factor (Fig 3). However our proteome analyses only identified 1636 proteins. Comparing these to PaxDB reported protein abundance levels [31] indicates our proteomics was largely limited to abundant proteins (>86% of those identified are among the top 40% abundant proteins), while our ‘core’ mRNA targets encode proteins that are predominantly of lower abundance (<20% are in the top 40% of proteins). As a consequence, very few of our identified core mRNA targets were quantified in our proteomics study (62/526, including 9 of the 4E-IND subset). 21 of the 62 revealed a statistically significant upregulation in at least one of the proteomics experiments, including 3 4E-IND mRNAs, consistent with relief of translational repression on these transcripts following loss or mutation of the 4E-BPs. As direct comparisons between mRNA targets and the proteome was complicated by limited proteome coverage, we decided to examine the RNA targets in more detail.

### Caf20p and Eap1p regulate mRNAs from diverse cellular pathways

**Fig 5:**
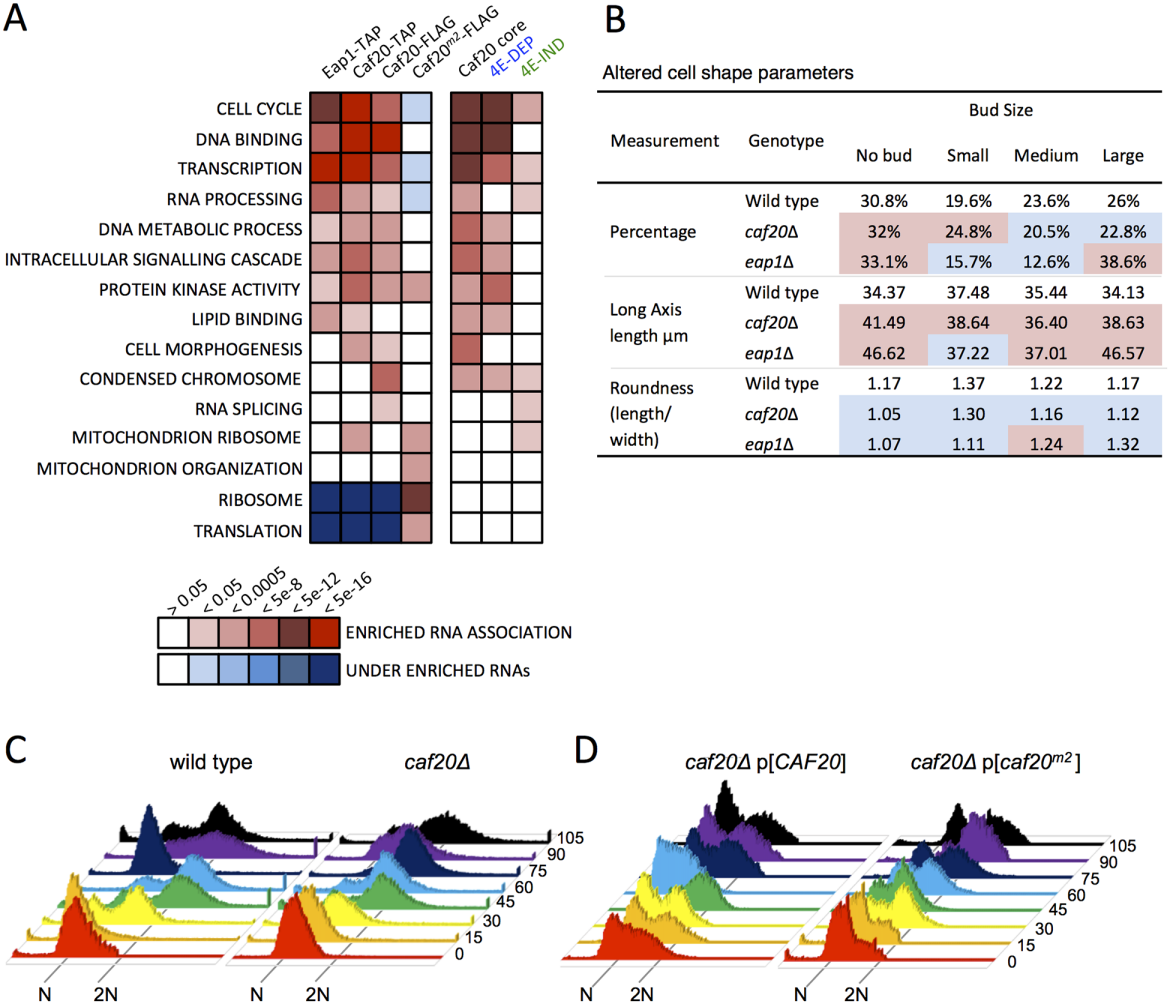
4E-BP-bound mRNAs enrich cell cycle and transcription factors. A. Functional GO class analysis of mRNAs associated with the 4E-BPs by RNA-seq. Over/underrepresentation colours depict FDR significance as per the key. Only a representative selection of the significant functional categories is shown. B. Summary of morphology changes noted within the *Saccharomyces cerevisiae* Morphological database (SCMD; http://yeast.gi.k.u-tokyo.ac.jp/datamine/) for 4E-BP deletion strains. Red/blue shading indicate over/under enrichment compared to wild type cells. C. D. Cell cycle progression defects in *caf20* mutants. Flow cytometry analysis of DNA content using Sytox Green staining is shown at the indicated times (min) following release from alpha factor arrest for C. wild type and mutated cells, and D. plasmid complemented mutant cells.

Functional analysis of the RNAs associating with Caf20p and Eap1p highlighted a diverse set of essential cellular pathways. These pathways ranged from ‘Cell Cycle’, ‘Transcription’ and ‘RNA processing’ to ‘DNA and lipid binding’, ‘intracellular signalling cascades’ and ‘cell morphogenesis’ (Fig 5A). These categories were also found to be overrepresented in RNAs whose association with polysomes increased in *caf20Δ* and *eap1Δ* cells [19]. An association between altered cell morphology and the 4E-BPs has been further reported in other studies [14,26]. All categories shared between the TAP-targets and Caf20-FLAG remain enriched in the core Caf20 targets, although the Caf20^m2^-FLAG targets differ significantly. They are enriched for mitochondrial and ribosome/translation targets, which are significantly under enriched in the wild type precipitates. These data suggest that having lost interaction with its principal partner eIF4E, Caf20^m2^p is binding other targets, chiefly those that normally encode abundant proteins. Repression of mitochondrial proteins was noted in our proteomics, providing a correlation between mRNA targets and translational repression (Fig 3). Another class shared between the proteomic changes and RNA targets is the cell cycle. This would suggest that there are genuine targets for translation repression and agrees with the observed enriched protein levels from the proteome analysis (Fig 3B).

Associations between the 4E-BPs and mRNAs important for the cell cycle has been postulated previously through regulation of the G_1_ cyclin *CLN3* mRNA, which can be regulated by mutating eIF4E or altering eIF4E levels [32,33] and through disruption of *PAB1* function by preventing formation of a closed loop [34,35]. The 4E-BPs have also been implicated in spindle pole body regulation [36] and *eap1* mutants have reported ploidy defects [35]. Our data, in conjunction with prior microarray analysis [19] would suggest that the 4E-BPs regulate genes important for cell cycle progression at multiple points, as they associate with mRNAs involved in the G_1_, S, G_2_, and M phases. In addition a systematic analysis of yeast deletion collection mutants has revealed that each 4E-BP mutant strain has significant morphology changes (*Saccharomyces cerevisiae* Morphological Database, http://scmd.gi.k.u-tokyo.ac.jp). Both mutants have larger, rounder cells and have an increased proportion of unbudded cells among other defects (summarised in Fig 5B).

To examine cell cycle defects in more detail, we arrested haploid cells with alpha mating factor to synchronise them in G1, then washed out the inhibitor to release cells into the cell cycle. Cell samples were harvested at 15 min intervals and their DNA content was analysed by flow cytometry following Sytox Green staining (Fig 5C). *caf20Δ* cells revealed a modest delay in initiating DNA replication—compare the 30 min samples where the majority of wild-type cells appear in S-phase (53%), whereas only 15% of *caf20Δ* cells have entered S-phase at this time (yellow in Fig 5C). A *caf20Δ* G2/M delay is also evident. By 75 min most wild type cells have returned to 1N (82%), whilst *caf20Δ* cells persist longer in G2/M. By 90 min synchrony is lost as mother and daughter cells have differing G1 phases.

The *caf20*Δ defects were not completely rescued by plasmid borne *CAF20* (Fig 5D, left). It is not clear if this is caused by aberrant plasmid segregation in some cells, or caused by the need for high alpha factor concentrations to induce cell synchrony the BY4741 strain background used throughout this study. In any event this complicates data analysis. The p[*CAF20*] cells appear to accelerate through G2/M faster than wild type as by 60 min ~86% of cells have returned to G1. Cells bearing caf20^*m2*^p appear to correct the G1-S delay seen in *caf20Δ* cells, but in contrast do not correct the G2/M-G1 transition (Fig 5D, right). At the 30 min and onwards time points analysed, the majority (>60%) of cells appear to be in G2/M. Our interpretation of these data are that Caf20 is required for a normal progression through the cell cycle and that Caf20^*m2*^ can restore some Caf20p functions in this assay suggesting that cell cycle defects are only partially dependent on the eIF4E interaction motif. This data agrees with the GO functional enrichments for cell cycle annotated genes (Fig 5A) and provides additional support for the idea that Caf20p has eIF4E-dependent and independent functions.

### Caf20p represses translation of its target mRNAs

**Fig 6:**
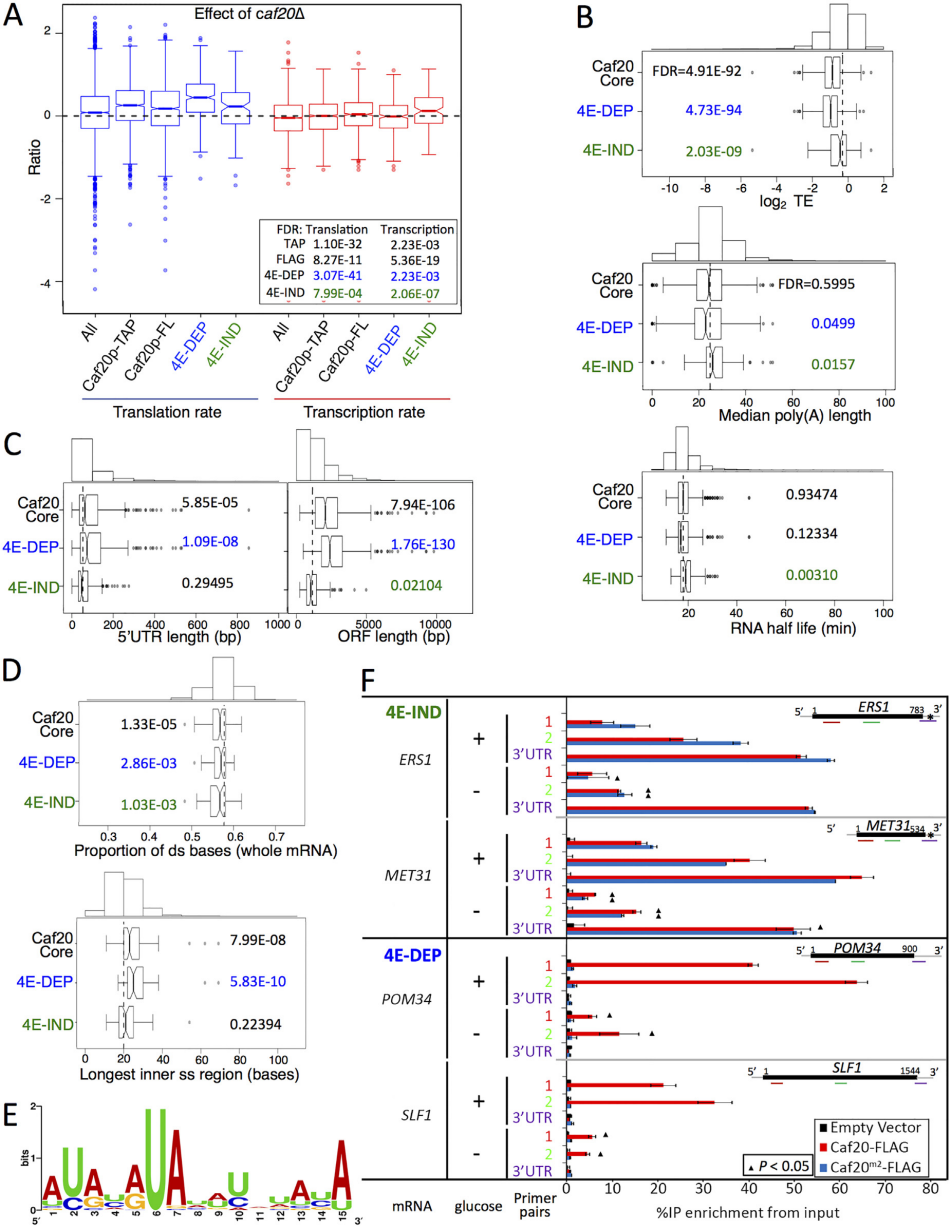
Features of Caf20p 4E-DEP and 4E-IND core bound mRNAs. A. Box and whisker plot showing impact of *caf20*Δ on polysome association (blue) and transcript abundance (red) across sets of Caf20p-associated mRNAs [19]. A 95% confidence interval around the median is represented by a notch. Where notches do not overlap the medians are different. Inset: *P* values represent FDR (Mann-Whitney U tests) versus non-target RNAs. B-D: Box and whisker plots comparing mRNA features of Caf20 core mRNA targets and the 4E-DEP and 4E-IND subgroups. Histograms above each plot show binned total data with the vertical dashed line indicating the median of the total. *P* values represent FDR (Mann-Whitney U tests corrected for multiple hypothesis testing). Gene sets were statistically tested versus the set of mRNAs not bound to TAP or FLAG tagged Caf20p. B. Translation efficiency (TE) data was taken from ribosome footprinting experiments, median poly A tail length [37] and mRNA half-life [38]. C. 5’ UTR and ORF lengths and D. secondary structure information [40] are similarly shown. E. Sequence motif identified in 3’UTRs of 4E-IND mRNAs using the MEME Suite [43]. See S1 Dataset, sheet 11 for individual motifs identified. F. Caf20p interactions with 4E-IND mRNA 3’UTRs and with ORFs differ in sensitivity to glucose starvation. Fraction of each indicated RNA (left) isolated in complex with Caf20-FLAG (red) or Caf20^*m2*^-FLAG (blue bars) or an empty vector control (black bars) from *caf20*Δ cells following formaldehyde cross-linking and RNase III digestion. qRT-PCR detection with primer pairs hybridizing along each RNA as indicated by colour coding in each cartoon (right). Samples were prepared from cells grown in SCD (+ glucose) or following 10 min of glucose starvation (- glucose). Statistically significant changes ± glucose indicated by ▲ (T-test).

We compared the mRNA targets of Caf20p with published datasets to determine common features and shared trends. Previously we performed translational profiling using microarrays to determine the ratio of individual mRNAs in polysomal or monosomal sucrose gradient fractions in wild type and *caf20*Δ cells [19]. Both Caf20-TAP and Caf20-FLAG associated mRNAs exhibit increased polysome association in *caf20*Δ cells (Fig 6A). These data are entirely consistent with Caf20p acting as a repressor of translation on its bound mRNAs. Both the 4E-DEP and 4E-IND subsets of the core binding set showed significant shift, with 4E-DEP targets shifting most, suggesting that Caf20p can repress translation via both eIF4E-dependent and-independent mechanisms. We also compared our gene sets against ribosome occupancy data from ribosome profiling experiments performed with wild type cells and using the ribosome footprinting technique [37]. It is clear that both 4E-DEP and 4E-IND core Caf20p mRNA targets have significantly lower ribosome occupancy, or translational efficiency, than non-Caf20p targets (Fig 6B), again 4E-DEP targets are most repressed. These trends are also seen in the larger data sets from each sequencing experiment (S3 Fig). Taken together both comparisons suggest that Caf20p has a strong repressive effect on the translation of over 500 core mRNA targets and may also contribute to reduced translation of a wider set of mRNAs.

Prior work also implicates Caf20p in RNA-decay [18,25,26]. Fig 6A (right plots) indicates that 4E-IND transcript levels increase most in *caf20Δ* cells, which is consistent with increased mRNA stability following *caf20*Δ. However this group also has longer median PolyA tail length and longer mRNA half-lives than non-Caf20p targets when compared with global dataset measurements in wild type cells, which does not support the suggestion Caf20p-binding generally promotes mRNA instability of these targets (Fig 6B) [37,38]. In contrast 4E-DEP mRNAs do have shorter polyA tails and shorter half-lives, although the latter was not deemed statistically significant (Fig 6B). Translational repression often precedes mRNA decay; hence in our view, these data comparisons provide stronger evidence for roles of Caf20p in translational repression than for a widespread direct role in RNA-decay.

### Shared structural features of Caf20p target mRNAs

We undertook a range of comparisons to identify common features of Caf20p target mRNAs. We found that core 4E-DEP mRNAs have longer 5’ UTRs and longer ORFs than non-Caf20p targets (Fig 6C). It has previously been observed that shorter ORFs are frequently associated with high translation efficiencies while longer ORFs have lower ribosome densities [39]. Association of Caf20p with longer mRNAs could help explain lower initiation rates and lower overall translation of these mRNAs. In contrast 4E-IND targets have shorter coding regions on average even than non-targets, which further differentiates the two groups of core Caf20p targets. A recent study examined RNA structure for the yeast transcriptome [40], an approach called parallel analysis of RNA structure or PARS. The core Caf20p target mRNAs have lower secondary structure overall, especially within the ORF, with 4E-DEP mRNAs possessing longer internal single stranded stretches (Fig 6D and S3 and S4 Figs). The affinity of eIF4E for the 5’ cap structure can be influenced by secondary structures close to the cap and also its interaction with binding partners such as eIF4G [41]. It has also been suggested, based on PARS analysis, that RNA structure close to the 5’ end of mRNAs is a factor important for determining Caf20p binding [42]. Comparing the core 4E-DEP and 4E-IND datasets with the PARS study does not indicate any statistically significant increased secondary structure of the 5’ cap proximal 30 nucleotides or elsewhere in the 5’ or 3’ UTRs.

We screened 5’ and 3’ UTRs of the core target mRNAs for the presence of shared sequence motifs using MEME [43]. No clear motifs were found within the 4E-DEP mRNAs. In contrast a motif was identified within the 3’ UTRs of the 4E-IND mRNA targets (Fig 6E and S1 Dataset, sheet 11). This 15 ribonucleotide motif is rich in A and U residues, predominately AU repeats or alternating pyrimidine and purine ribonucleotides. The motif does not correspond with any known RNA-binding motif [44], It is found in some non-target 3’UTRs, but it is extremely rare in 5’UTRs. One possibility is that Caf20p directly binds to this mRNA sequence. *CAF20* mRNA contains the motif in its 3’ UTR (S1 Dataset, sheet 11).

### Caf20p binds to ORFs and to 3’UTRs of 4E-IND mRNAs

We thought it possible that Caf20p binding to 3’UTRs might contribute to 4E-IND mRNA binding. To assess this, we devised a qRT-PCR assay to assess Caf20p and Caf20^*m2*^p binding to ORF and 3’UTR regions of selected 4E-DEP and 4E-IND mRNAs. Our approach included formaldehyde cross-linking of *caf20Δ* cells containing a vector, Caf20-FLAG or Caf20^*m2*^-FLAG, prior to capture of the proteins on FLAG affinity resin. Captured associated RNA was digested with Rnase III to fragment it, but retain RNA of sufficient size to permit amplification by PCR. Fragmented RNAs were converted to cDNA for qPCR. Our first set of experiments were performed with primers to *CAF20* and three additional 4E-IND mRNAs bearing a 3’ UTR motif (*ERS1*, *YPR017W*, *MET31*) and three 4E-DEP RNAs with no 3’ UTR motif (*POM34*, *SLF1*, *SNF1*). For each RNA we used primer pairs within the coding and 3’ UTR regions encompassing the putative 3’ UTR motif, where present. Each 4E-DEP mRNA assessed was enriched with Caf20-FLAG within the coding regions, but not 3’UTRs (S5 Fig). In contrast binding to Caf20^*m2*^-FLAG was greatly diminished within coding regions and remained low within 3’UTRs. This is consistent with the idea that both eIF4E interaction at the 5’ cap and ribosome binding is important for Caf20p to associate stably with these mRNAs and implies that interactions with eIF4E are critical for stable binding of Caf20p to these mRNAs. Strikingly both coding region and 3’ UTR regions of 4E-IND mRNAs bound to both wild type and mutant Caf20p (S5 Fig). Furthermore, for each mRNA tested, interaction with Caf20p was highest for the probes in 3’UTRs. The data provide strong support to the idea of separate 4E-DEP and 4E-IND classes of Caf20 interacting RNAs.

To assess whether any of the mRNA interactions identified were dependent upon ribosomes, we stressed cultures by removing glucose for 10 min prior to cross-linking. Glucose withdrawal causes a well characterised ribosome run-off [45] (Fig 2D). The tested 4E-DEP and 4E-IND mRNAs behaved comparably in non-starved cells between the two independent experiments (compare + Glucose in Fig 6F with S5 Fig). However when glucose was removed Caf20p binding to coding regions was greatly diminished. This data strongly suggests that interactions between Caf20p and mRNA coding regions is dependent on translating ribosome interactions. In marked contrast binding to 3’UTRs of the 4E-IND mRNAs *ERS1* and *MET31* was maintained. These data are consistent with the idea that 3’ UTR interactions can contribute to Caf20p recruitment to specific mRNAs even when eIF4E interactions are prevented, suggesting that 3’ UTR binding combined with ribosome interactions contribute to eIF4E-independent recruitment of the 4E-BP Caf20p to specific mRNAs.

**Fig 7:**
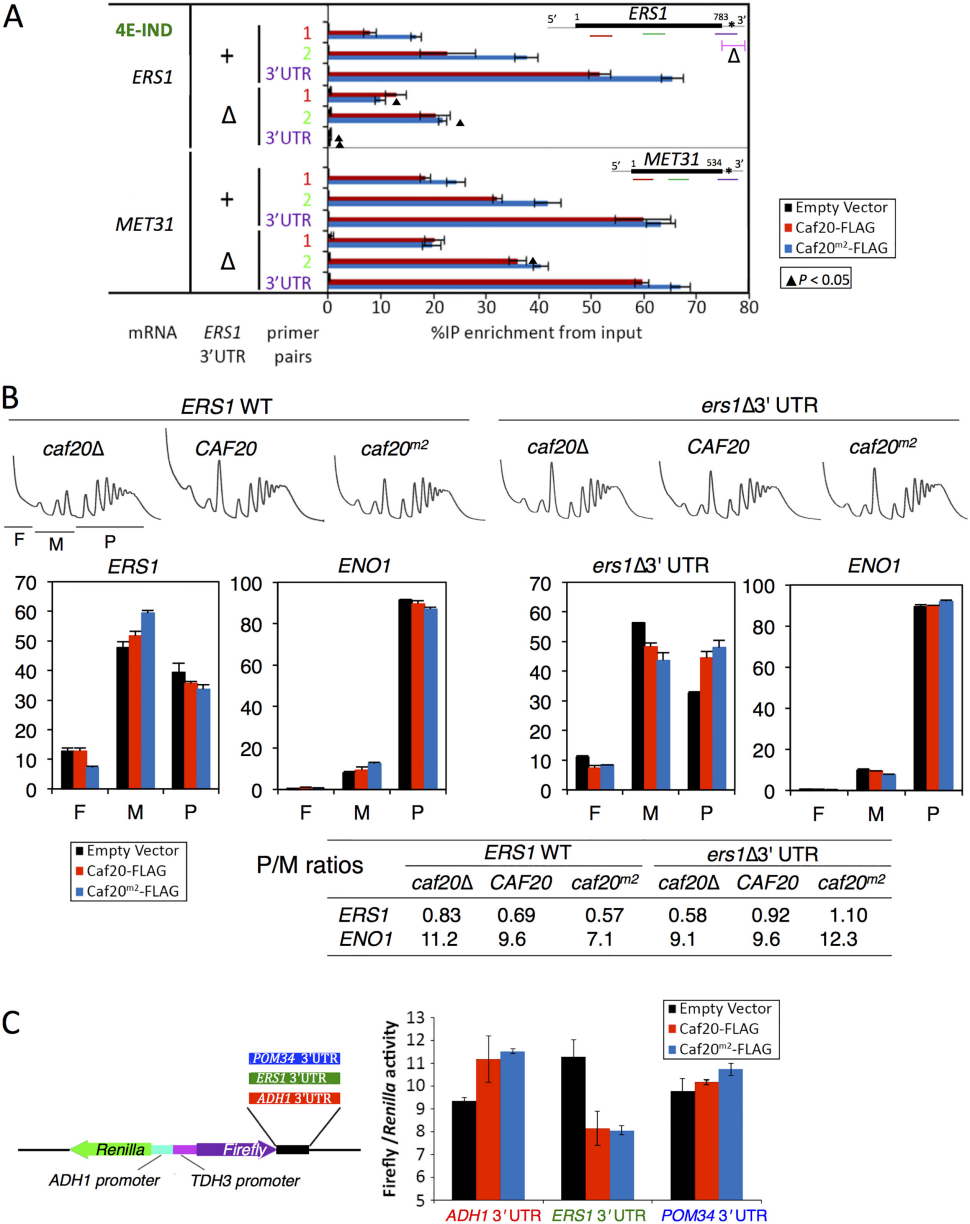
Caf20p and *ERS1* 3’UTR contribute to the translational repression of *ERS1*. A. Interactions between Caf20^*m2*^-FLAG and *ERS1* is diminished by loss of *ERS1* 3’UTR. Fraction of *ERS1* or *MET31* RNA (left) isolated in complex with Caf20-FLAG (red) or Caf20^*m2*^-FLAG (blue bars) or an empty vector control (black bars) from *caf20*Δ cells following formaldehyde cross-linking and RNase III digestion. qRT-PCR detection with primer pairs hybridizing along each RNA as indicated by colour coding in each cartoon (right). Statistically significant changes ± *ERS1* 3’UTR indicated by ▲ (T-test). B. Polysome engagement of *ERS1* is affected by Caf20p and the *ERS1* 3’UTR. Polysome profiles from sucrose gradient analyses of strains are shown (top) with free (F), monosomes (M) and polysome (P) regions indicated beneath the *caf20*Δ profile. RNA collected from sucrose gradient fractions was analysed by qRT-PCR and quantified relative to a luciferase RNA ‘spike-in’ control. Proportions of RNA in ribosome free (F), monosomes (M) or polysome (P) are shown for *ERS1* and *ENO1*. Bar chart colors are as in A. P/M ratios for both mRNAs calculated from the data are indicated in the table below. C. *ERS1* 3’UTR confers Caf20p-dependent repression to firefly luciferase. Cartoon of plasmid dual luciferase construct and cloned 3’UTR fragments (left). Ratio of firefly to *Renilla* luciferase activities for transformants of *caf20*Δ cells bearing indicated Caf20 plasmid. Mean ±SE for triplicate assays (right).

To further test these ideas we focussed our attention on *ERS1*, one of the 4E-IND targets studied in Fig 6F. We replaced its 3’UTR by homologous recombination with a synthetic sequence from pYM19, one of a popular series of genome integrating carboxy-terminal tagging vectors [46] to create a series of strains where we could assess the impact of both Caf20p variants and the *ERS1* 3’UTR. Repeating our Caf20p-FLAG IP and mRNA cross-linking assay revealed that loss of the authentic 3’UTR from *ERS1* resulted in reduced interaction between Caf20^*m2*^-FLAG and *ERS1* coding sequences (compare *ERS1* bars marked Δ with + in Fig 7A). *ERS1* coding sequence interactions with Caf20^*m2*^-FLAG were diminished to the level of binding seen with WT Caf20-FLAG, likely reflecting retained ribosome-mediated binding. 3’UTR binding was eliminated confirming these sequences have been removed. In contrast and as expected the interactions between WT or mutant Caf20p and *MET31*, a control mRNA not mutated, remained unaffected by replacement of the *ERS1* 3’UTR (Fig 7A).

To assess the impact of the 3’UTR on the translatability of *ERS1* as well as its responsiveness to Caf20p, we examined the impact of Caf20p and the *ERS1* Δ3’UTR on the migration of *ERS1* and a control RNA, *ENO1*, across polysome gradients by qRT-PCR. *ENO1* mRNA does not interact with Caf20-FLAG or Caf20^*m2*^-FLAG (Fig 4B). ENO1 mRNA is very well engaged with polysomes (~90% of the mRNA is in polysomal fractions), and this appears largely unaffected by Caf20p or *ERS1* status, as expected (Fig 7B). In contrast *ERS1* mRNA is poorly engaged with polysomes (<40% in polysomes), which is indicative of translational repression at the initiation phase. Caf20p appears to contribute to translational repression, but is likely not the sole regulator because *ERS1* mRNA remains predominantly monosomal in *caf20*Δ cells (Fig 7B, left). Replacing *ERS1* 3’UTR reduced polysome association in *caf20*Δ cells, but enhanced it in Caf20p and Caf20^*m2*^p cells (*ers1*Δ 3’UTR, Fig 7B). Together these data suggest that Caf20p binding to *ERS1* 3’UTR can contribute to its translational control, independently of the protein’s ability to bind eIF4E.

Finally to assess if a Caf20p-responsive 3’UTR was sufficient to confer Caf20-dependent repression, we cloned the *ERS1* 3’UTR downstream of the firefly luciferase ORF in a dual firefly-*Renilla* reporter plasmid [47]. As controls we used the parent vector (pTH727) which has an *ADH1* 3’UTR that is not bound by Caf20p in any of our experiments and the *POM34* 3’UTR (Fig 7C). As *POM34* is an eIF4E-dependent target of Caf20p, its 3’UTR does not bind Caf20p (Fig 6F). Dual luciferase assays were done in *caf20*Δ cells co-transformed with plasmid expressing Caf20-FLAG, Caf20^m2^-FLAG or vector alone. Strikingly these experiments revealed that the *ERS1* 3’UTR confers Caf20p-dependent repression of firefly luciferase expression, while neither control 3’UTR behaved this way (Fig 7C). These data are consistent with the idea that Caf20p interactions with specific 3’UTRs can confer eIF4E-independent repression of a heterologous gene. Together these experiments characterize a novel eIF4E-independent translational control mechanism for the 4E-BP Caf20p.

## Discussion

In this study we set out to identify protein and RNA-binding partners for Caf20p and Eap1p, with the aim of furthering our understanding of their scope and mechanisms of translational control. We find that in addition to eIF4E, the 4E-BPs interact with intact ribosomes and polysomes (Figs 1 and 2). The 4E-BPs are not core ribosomal proteins as they behave like other associated factors that can be disrupted by increased salt or removal of magnesium (Fig 2 and S1 Fig). We also identified several candidate RNA-binding proteins that may contribute to control. Unlike the ribosome and eIF4E interactions, these protein interactions were destabilized by RNase treatment (Fig 1E), so were considered indirect interactions and not considered further. 4E-BPs can bind to ribosomes independent of their interaction with eIF4E because Caf20^*m2*^p and Eap1^*m3*^p retain ribosome interactions (Fig 2 and S1 Fig), but not eIF4E-binding. Additionally both Caf20^*m2*^p and Eap1^*m3*^p retain physiologically important functions, as expression of the mutant proteins each suppresses the respective *caf20*Δ and *eap1*Δ slow-growth phenotypes observed on glycerol-ethanol medium as well as the native proteins (Fig 3C) and because Caf20^*m2*^p partially suppressed cell cycle progression defects (Fig 5D). In addition our proteomics study indicates that expression levels of many of the proteins we could quantify that differed between *caf20*Δ and WT cells were corrected in Caf20^*m2*^p cells (Fig 3). Together these data suggest that at least some 4E-BP functions do not require eIF4E-binding. An eIF4E-independent function has previously been suggested for Eap1p as a mutant in the eIF4E-binding motif, eap1-Y109A, complemented a temperature dependent genetic instability phenotype [35]. However the mutant used was later shown to retain residual eIF4E binding [14] and as we show here should retain ribosome interactions (S1D Fig). As Caf20^*m2*^p also maintains 80S binding, our data suggests a role for ribosome interaction in eIF4E-independent function.

Unfortunately, our attempts to define the ribosome-binding motif in Caf20p were not successful. The eIF4E-interacting motif is at its extreme N-terminus. In common with many 4E-BPs from higher eukaryotes the 161 residue Caf20p protein has no clear domains or structure, but has alternating positively and negatively charged regions [42]. Sequence comparisons across Caf20p proteins in related yeasts identified the acidic C-terminus as conserved. In addition we detected a faster migrating form of Caf20p that consistently migrated away from ribosomes (see for example Fig 2). Extensive investigations failed to identify the cause of this faster migrating species, which retained eIF4E binding and was not caused by altered phosphorylation. Finally, specifically deleting the acidic C-terminus failed to disrupt ribosome binding and so the ribosome-binding region within Caf20p remains undetermined.

In a recent study we used RNA-Sequencing of mRNAs associated with TAP-tagged factors to identify relative affinities of mRNAs for eIF4E, eIF4GI, eIF4G2, Pab1p, Caf20p and Eap1p [20]. That study revealed distinct classes of mRNAs that exhibit different associations with each translation factor. Two of these classes were enriched for Caf20p and/or Eap1p binding. There was a broad overlap between the identity of Caf20p and Eap1p-bound transcripts, and also between eIF4E and eIF4G1 and eIF4G2, but much poorer correlations between the 4E-BPs and the other factors [20]. Here we have extended our analyses of 4E-BP target mRNAs by performing additional sequencing of Caf20p-targets using an alternative FLAG-affinity purification scheme and a complementary sequencing platform (Illumina HiSeq versus our prior ABi SOLiD 4 analysis). Both Caf20p datasets overlap well enabling us to identify a core set of 526 Caf20p-mRNA targets identified with high reproducibility across both platforms (Fig 4). Our new experiments also identified that ~25% of these core mRNA targets bind to Caf20p independently of the Caf20p-eIF4E interaction (Fig 4). By two criteria, both 4E-DEP and 4E-IND targets appear translationally repressed by Caf20p; the targets have low ribosome occupancy in wild type cells and the mRNAs migrate more into polysomes upon *CAF20* deletion (Fig 6).

**Fig 8:**
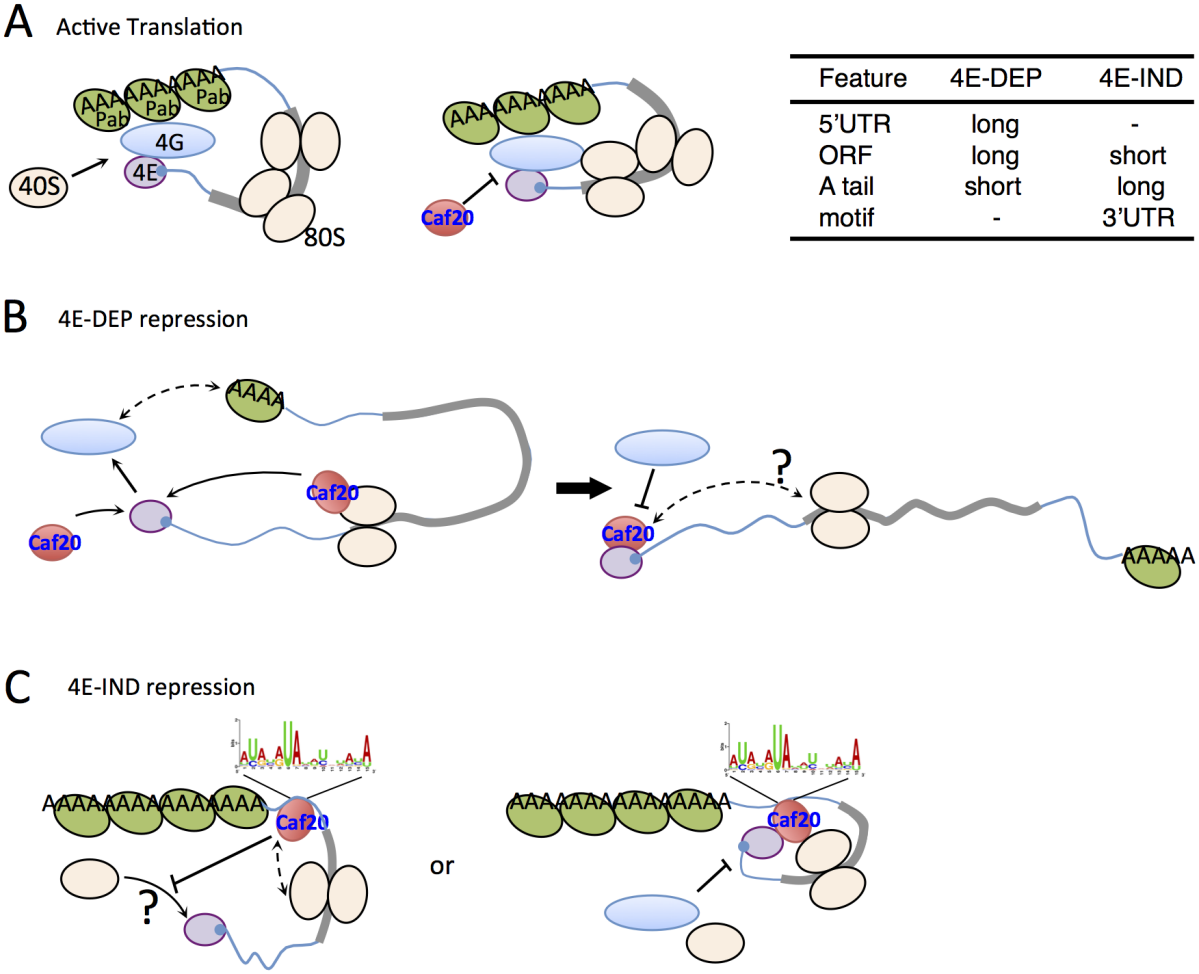
Models for 4E-DEP and 4E-IND Caf20p-mediated translational repression. A. Active translation. Short mRNA (blue line with grey ORF) shown with eIF4E (purple) bound to 5’cap (blue), eIF4G (blue oval) and Pab1p (green oval) facilitating mRNA circularisation and ribosome recruitment (cream ovals) that excludes Caf20p binding eIF4E. B. Caf20p mediated eIF4E-dependent translational repression. eIF4E/4G/Pab1p closed loop unstable on long mRNAs facilitating Caf20p binding (red). Caf20p binding to 80S ribosomes may contribute to its local recruitment. C. 4E-IND repression mechanisms. Caf20p 3’ UTR motif binding (may not be direct), either alone or in addition to 80S binding facilitates translational repression. eIF4E-Caf20p binding may contribute to repression on some of these mRNAs.

The classic model of 4E-BP mediated translational repression from mammalian 4E-BP1 invokes direct competition between eIF4G and 4E-BP1 for a shared binding motif on eIF4E to attenuate translation initiation [8]. Caf20p and yeast eIF4G1 and eIF4G2 all similarly share an overlapping interface with eIF4E [48]. However the motif alone cannot provide mRNA specificity to the repression that we have observed in terms of preferred mRNA binding targets. One alternate possibility is that sequence or structure-specific RNA-binding protein partners contribute to 4E-BP mRNA binding as observed for several metazoan proteins and also proposed previously for Eap1p [8,36]. However comparing our core Caf20p target mRNA identities with those of mRNA-binding proteins identified by others [44] failed to identify any significant overlaps. In addition our proteomics analyses failed to identify any new direct protein-interacting partners for Caf20p with the exception of the 80S ribosome (Figs 1 and 2). Our mRNA target analyses suggest that core 4E-DEP Caf20p interacting mRNAs possess both longer 5’UTRs and longer ORFs, with lower than average secondary structure, but have shorter polyA tails and a shorter mRNA half-life than non-Caf20p targets (Fig 6). The eIF4E/4G/Pab1p ‘closed loop’ complex is more stable when formed on short mRNAs with longer polyA tails in *in vitro* translation extracts [49]. In agreement with this idea we recently showed that eIF4E/4GI/4GII complexes are enriched on shorter mRNAs [20]. These observations are consistent with a model where eIF4E/4G interactions are stabilized by Pab1p and mRNA circularization on short mRNAs, but that this complex is less stable on longer mRNAs with shorter polyA tails allowing Caf20p to displace eIF4G and repress translation. This provides a simple model for eIF4E-dependent Caf20p mediated repression (Fig 8). As a significant fraction of Caf20p binds to translating ribosomes (Fig 2) it seems feasible that this could provide a local reservoir of Caf20p to facilitate rapid eIF4E binding following any disruption of the eIF4E-eIF4G interaction. This idea is depicted in Fig 8B. The experiments shown in Fig 6F indicate that ribosome binding by Caf20p may help stabilize its interactions with 4E-DEP mRNAs. Following a ten-minute glucose starvation causing ribosome run-off, binding of Caf20p to the tested 4E-DEP mRNAs was significantly reduced.

As with 4E-DEP targets, 4E-IND targets are also relatively poorly translated in wild type cells and have enhanced polysome association in *caf20*Δ cells (Fig 6A and 6B), consistent with Caf20p repressing their translation initiation. Unlike 4E-DEP mRNAs, 4E-IND targets have long polyA tails and longer median half-lives, do not have long 5’UTRs and have very short ORFs (Fig 6B and 6C). With the notable exception of low translation efficiency, these mRNA features are commonly found in highly translated mRNAs. The data suggested that an additional feature must be necessary for effective Caf20p recruitment. A candidate we have identified is the 3’ UTR, in which a conserved AU-rich motif found enriched in 4E-IND targets (Fig 6E). We show that 3’UTRs can facilitate stable Caf20p binding (Fig 6F and S5 Fig) even following glucose starvation, implying that 3’UTR binding to 4E-IND targets is neither ribosome or eIF4E-mediated and that the 3’UTR can stabilize Caf20p binding to the tested mRNAs. Similar interactions were not seen with 4E-DEP mRNAs tested and are consistent with ribosome profiling experiments where ribosomes do not normally associate with 3’UTRs. The data therefore suggest that 4E-IND 3’UTRs contribute to mRNA-specific recruitment of Caf20p to facilitate 4E-IND translational control. To test this directly we focussed on *ERS1* mRNA. We found that swapping the *ERS1* 3’UTR for a heterologous sequence weakened Caf20^*m2*^p binding (Fig 7A) and relieved some translational repression, as determined by altered mRNA migration in polysome gradients (Fig 7B). These data are consistent with our idea that Caf20p binding the *ERS1* 3’UTR contributes to repression of its translation initiation. We also found that the *ERS1* 3’ UTR is able to confer Caf20p-dependent repression of translation to a heterologous reporter gene (Fig 7C). Although we have not directly demonstrated a role for the identified AU-rich motif, our data is consistent with the idea that specific 3’UTRs can contribute to Caf20p recruitment and this is shown in the models in Fig 8C.

How Caf20p mediates translational repression in an eIF4E independent manner is not yet clear. As 4E-IND mRNAs have low ribosome occupancy (Fig 6B), this is consistent with a role for repression of translation initiation. Repression solely at elongation, slowing ribosome migration rates would normally be expected to increase ribosome densities. It is possible that initiation-mediated translation repression could still involve eIF4E-interactions, although this is unlikely to account for all Caf20p activities because *caf20*Δ cell growth defects on glycerol-ethanol medium are suppressed by Caf20^*m2*^p (Fig 2E). Our data do not provide evidence for competition between eIF4E and ribosomes for Caf20p binding, because both wild type and mutant Caf20 proteins seem equally capable of binding ribosomes. In Fig 2F Caf20^*m2*^p appears slightly enriched in sub-polysomal fractions (lanes 4–8) than the wild-type protein, but the significance of this is not clear. Addressing the detailed mechanisms at work will require experiments beyond the scope of this study. In summary, this work has provided evidence that Caf20p regulates translation of long mRNAs encoding larger proteins via the classic eIF4E-binding motif. In addition it can repress translation of shorter transcripts via a novel mechanism that likely involves ribosome binding and a 3’ UTR element. We also show that disruption of Caf20p or Eap1p functions causes broad changes to the proteome that extend beyond their immediate mRNA targets (Fig 3). This work extends the capacity for Caf20p to regulate translation beyond its known role in cap-dependent inhibition.

## Materials and Methods

### Strains and growth conditions

Yeast strains used in this study are listed in S1 Table. TAP-tagged His^+^ strains in the BY4741 background were obtained from Open Biosystems. An untagged *HIS3* BY4741 control strain (GP6001) was used as a control for all TAP experiments [20]. Strains GP6691, GP6692 and GP6693 were generated by transforming BY4741 *caf20*Δ::KanMX (GP6320) (Euroscarf) with a control vector pRS426 [*URA3* 2*μ*]; pAV2337 [*CAF20*-FLAG_2_ *URA3* 2μ] a plasmid expressing Caf20p with tandem C-terminal FLAG Tags (synthesised by Epoch Life Sciences); or plasmid pAV2338 [*CAF20*-Y4A,L9A-FLAG_2_ *URA3* 2μ] expressing the Y4A,L9A double mutation that disrupts 4E-binding (termed *caf20* ^*m2*^) [14]. Strains GP5145 and GP5148 were generated by insertion of pJF3898 [HA-*EAP1 LEU2 CEN*] and pBMK492 [HA-*EAP1* ^*m3*^ *LEU2 CEN*] (bearing Y109A and L114A mutations and termed *eap1* ^*m3*^) [14] into a BY4741 *eap1Δ*::*KanMX* strain. Single and double 4E-BP deletions strains GP6801-5 used in the label free proteomics study were generated by mating and tetrad dissection as described previously [45]. For strains GP7021-3, the 3’UTR of *ERS1* was replaced by homologous recombination using a PCR fragment containing *HIS3MX6* from pYM19 and beginning downstream of the Myc tag. The new 3’UTR is artificial/vector derived and encoded by this commonly used vector series [46]. *CAF20* and *caf20* ^*m2*^ were subcloned from pRS316 vectors into pRS425 using SpeI and SalI to create pAV2421 and 2422. The *ERS1* and *POM34* 3’UTRs were amplified with primers listed in S2 Table and cloned into the dual luciferase vector pTH727 [47] using NotI and SalI to replace the *ADH1* 3’UTR downstream of firefly luciferase.

Strains were grown at 30°C in synthetic complete dextrose media lacking either histidine [SCD-his] (TAP affinity studies) or uracil [SCD-ura] (FLAG-tagged plasmid studies) [50]. To study growth in respiratory media, the carbon source was switched from 2% glucose to 3% ethanol, 1% glycerol (SCGE). Strains GP6834–GP6841, GP6846 and GP6847 were grown to an A_600_ of 0.2, pelleted, washed and resuspended in prewarmed fresh SCGE media and their growth rate assessed hourly. For flow cytometry the untransformed parent strains of GP6801-5 were mating type switched by transformation with p[*HO URA3*] and the resulting diploid cells tetrad dissected to obtain *MAT* **a** strains GP6991-2. Transformation with *CAF20* or *caf20* ^*m2*^ generated GP7010 and 7013.

### TAP affinity purifications

TAP-affinity purifications were performed as described previously [23]. Briefly, yeast cultures were grown to A_600_ = ~0.6, pelleted, snap frozen in liquid nitrogen and ground in Buffer A (20 mM Tris-HCl [pH 8], 140 mM NaCl, 1 mM MgCl_2_, 0.5% NP40, 0.5 mM DTT, 1 mM PMSF, EDTA free Protease Inhibitor cocktail tablet (Roche), 100 μM NaV_3_O_4_, 5 mM NaF and 40 units/ml RNAsin) using liquid nitrogen and a 6870 Freezer Mill (Spex). Lysates were cleared through two centrifugation steps (15,000 x *g* at 4°C) and quantified using Bradford Reagent. 10 mg total protein was loaded onto Rabbit IgG coupled Tosyl-activated Dynabeads M-280 magnetic beads (Dynal), with the exception that altered amounts of Eap1-TAP (25 mg) and Pab1-TAP (2 mg) extracts were used to ensure maximum binding of the tagged proteins. Extracts were incubated for 20 min at 4°C and then washed x5 with buffer A.

For protein identification, after the final wash, the beads were briefly washed with TAP peptide elution buffer (40mM Tris-HCl, pH 8; 100 mM NaCl, 1 mM EDTA, pH 8; 0.01% v/v Tween 20; 5% v/v EtOH) and then incubated in 250 μl TAP peptide elution buffer containing 2 mM TAP elution peptide [(PEG)4-DCAWHLGELVWCT cyclised by disulphide, where (PEG)4 is a 16 atom polyethylene glycol spacer (Peptide Protein Research Ltd., Fareham, UK)] for 15 min [24]. Eluted peptides were concentrated to 20 μl using Amicon Ultra 0.5 mL Filters with a 3 KDa molecular weight cut off (Millipore) and loaded onto a 12% SDS-PAGE resolving gel with no stacking gel. Samples, five replicates of each, were electrophoresed into the top 1 cm of the gel and stained by Instant Blue Stain (Expedeon Limited, Cambridge, UK). The immunoprecipitated proteins were excised from the gel, digested with trypsin and processed by the Michael Smith Biological Mass Spectrometry Facility (see LC-MS/MS below). For the reciprocal protein verification study, beads were re-suspended in 20 μl 1x SDS loading buffer following the final wash.

### Caf20-FLAG and Caf20^m2^-FLAG affinity purifications

Cell extracts were processed and quantified as described above. 10 mg total protein was loaded onto Anti-FLAG M2 Magnetic Beads (Sigma Aldrich Company Ltd., Dorset, UK) and incubated at 4°C for 2 h. The Anti-FLAG beads were then washed with Buffer A (3 x 1 min washes and 2 x 15 min washes). Following the final wash, proteins were eluted by incubation for 2 h in Buffer A containing 200 μg 3xFLAG Peptide. Protein eluates were concentrated to 20 μl using Amicon Ultra 0.5mL Filters with a 3 KDa cut off (Millipore, Billerica, MA, USA) and processed as above.

### Label-free protein quantification by LC-MS/MS

TAP and FLAG associated protein samples were prepared as above. For whole cell proteomics, strains were grown in SCD-URA to an A_600_ of 0.6. Cultures were harvested, snap frozen in liquid nitrogen and lysed using liquid nitrogen and a 6870 Freezer Mill (Spex). Cells were resuspended 25 mM ammonium bicarbonate buffer containing a protease inhibitor cocktail tablet (Roche). Five replicates were made for each strain. Lysates were defrosted and cleared by centrifugation at 4°C (15,000*g* for 10 min) and 100μg of cleared lysate was diluted to a final volume of 160μl containing 1% (w/v) RapiGest (Waters Corporation). Samples were incubated at 80°C for 10 min, 600 rpm, and reduced using a final concentration of 3.5 mM DTT in 25mM ammonium bicarbonate and incubated at 60°C for 10 min, 600 rpm. Cysteine residues were uniformly modified using iodoacetamide (IAM) to a final concentration of 10 mM and incubated at room temperature for 30 min. Samples were subsequently treated by two trypsin digestions (0.01 μg/μl trypsin in 10 mM acetic acid) for 4.5 h initially and then overnight at 37°C, 600 rpm. Hydrochloric acid was added to a final concentration of 13 mM in between the two digestions to maximise trypsin digestion. The Rapigest was then removed from the samples with the addition of 0.5 μl of trifluoric acid and incubation at 37°C for 2h followed by 7.5 μl of acetonitrile:water (2:1) and incubation at 4°C for 2h. Finally, samples were cleared by centrifugation at 13,000 *g* for 15 min. Supernatant was removed and desalted using OLIGO R3 reversed-phase media on a microplate system. Peptides were eluted in three cycles of 50% acetonitrile and dried by vacuum centrifugation, and reconstituted to 10 μl with 5% acetonitrile and 0.1% formic acid.

All digested samples were analysed by LC-MS/MS using an UltiMate 3000 Rapid Separation LC (RSLC, Dionex Corporation, Sunnyvale, CA) coupled to an Orbitrap Elite (Thermo Fisher Scientific, Waltham, MA) mass spectrometer. Peptide mixtures were separated using a gradient from 92% A (0.1% FA in water) and 8% B (0.1% FA in acetonitrile) to 33% B, in 44 min at 300 nl min^-1^, using a 250 mm x 75 μm i.d. 1.7 mM BEH C18, analytical column (Waters). Peptides were selected for fragmentation automatically by data dependant analysis.

The acquired MS data from the five replicates were analysed using Progenesis LC-MS (v4.1, Nonlinear Dynamics). The retention times in each sample were aligned using one LC-MS run as a reference, then the “Automatic Alignment” algorithim was used to create maximal overlay of the two-dimensional feature maps. Features with charges ≥ +5 were masked and excluded from further analyses, as were features with less than 3 isotope peaks. The resulting peak lists were searched against the *Saccharomyces* Genome Database (SGD, version 3^rd^ February 2011) using Mascot v2.4 (Matrix Science). Search parameters included a precursor tolerance of 5 ppm and a fragment tolerance of 0.5 Da. Enzyme specificity was set to trypsin and one missed cleavage was allowed. Carbamidomethyl modification of cysteine was set as a fixed modification while methionine oxidation was set to variable. The Mascot results were imported into Progenesis LC-MS for annotation of peptide peaks. The mass spectrometry proteomics data have been deposited to the ProteomeXchange Consortium [51] via the PRIDE partner repository with the dataset identifiers PXD001348 and DOI 10.6019/PXD001348 (whole cell shotgun proteomics), and PXD001407 and DOI 10.6019/PXD001407 (TAP-IP).

### Ribosome co-sedimentation analyses

Polyribosomal profiling was performed as previously described. Briefly, *S*. *cerevisiae* was grown to an A_600_ = 0.6, cycloheximide was added to a final concentration of 0.1 mg/ml and harvested by centrifugation. When cells were stressed with glucose starvation cultures were grown to an A_600_ 0.6, split into two 50 ml cultures, pelleted and re-suspended in either 50 ml SCD-HIS or SC-HIS for 10 min before addition of cycloheximide. *S*. *cerevisiae* were lysed into polyribosomal buffer containing cycloheximide [52] and 5 A_260_ units were loaded onto a sucrose gradient. 15–50% sucrose gradients were poured as previously described [52]. 5–25% sucrose gradients were poured in six separate fractions, increasing in 5% sucrose intervals. For the RNase I digestion, a polyribosome extract were generated as described above. The extract was split and either incubated with 20 U SUPERase•In (Life Technologies, Paisley, UK) or 10 U RNase I (Life Technologies, Paisley, UK) and 20 U SUPERase•In (Life Technologies, Paisley, UK) at 21°C for 30 min. A total of 14 x 1 ml fractions were collected across the sucrose gradients. Proteins were precipitated from the collected fractions with trichloroacetic acid (10% final concentration), washed twice with acetone and re-suspended in SDS loading buffer. RNA was extracted from the collected fractions using a standard Trizol Reagent (Life Technologies, Carlsbad, CA) protocol and res-suspended in 20 μl DEPC treated water. Sucrose cushion gradients were performed as described previously [53].

### Western blot analyses

Protein samples were mixed with 2 x SDS loading dye and heated to 95°C for 10 min to dissociate protein complexes from the IgG Tosyl-activated Dynabeads M-280 magnetic beads or Anti-FLAG M2 Magnetic Beads. IP samples were resolved by SDS—PAGE, electroblotted onto nitrocellulose membrane and probed using the relevant primary antibody. TAP tagged proteins were detected using an HRP-conjugated primary antibody to Protein A (Abcam, Cambridge, MA). All other primary antibodies were detected with horseradish peroxidase (HRP)-conjugated rabbit secondary antibody, with the exception of the Pab1p, which was detected using HRP-conjugated mouse secondary antibody.

### Alpha factor arrest and flow cytometry

Method was adapted from [50]. Cells from overnight cultures were used to inoculate SC medium containing 50 mM filter-sterilised sodium succinate to A_600_ = 0.1 and cultures were grown at 30°C to A_600_ = 0.3 before alpha mating factor (Sigma) was added to 10 μg/ml. Cells were incubated at 30°C and monitored microscopically for shmoo formation (~3–4 h). Cells were pelleted and washed twice in prewarmed SC medium to release from arrest. Samples for flow cytometry analysis of DNA content were taken at indicated times, fixed with 70% ethanol, treated with RNaseA and stained with 1 μM Sytox Green (Life Technologies), sonicated (Branson Sonifier 150) and DNA content determined on a Cyan ADP flow cytometer (Beckman Coulter) [50].

### RNA sequencing

Caf20-FLAG associated RNA was isolated on M2 magnetic beads as described above. For RNA target identification, after the final wash, the beads were resuspended in 270 μl Buffer A. A 20 μl aliquot of the sample was set aside for western blot analysis, and RNA was purified from the remaining 250 μl. Total RNA and IP RNA were isolated using a standard Trizol Reagent (Life Technologies, Carlsbad, CA) protocol and re-suspended in 10μl DEPC treated water as described previously [23]. RNA was quantified using a Nanodrop 8000 spectrophotometer (Thermo Fisher Scientific, Waltham, MA).

Total RNA samples were normalised to the amount of RNA isolated from the corresponding IP sample. rRNA was then depleted from the RNA samples using the Ribo-Zero^TM^ Magnetic Gold Kit (Yeast) for RNA-Seq (Epicentre, Madison, WI 53719), ethanol precipitated and re-suspended in 10μl DEPC water following the manufacturers guidelines. rRNA depletion was checked on a 2100 Bioanalyzer (Agilent Technologies, Palo Alto, CA) using a RNA nano chip and the remaining RNA stored at -80°C. Sequencing libraries were then generated using the TruSeq Stranded mRNA sample preparation guide from Illumina with 5μl of the ribosome depleted RNA added to 13μl of the fragment prime and finish mix. Libraries were validated on an Agilent Technologies 2100 Bioanalyzer, using the Agilent DNA 1000 chip to confirm the size (~260 bp) and purity of the sample. Samples were run on an Illumina HiSeq 2000. Illumina HiSeq reads were mapped to the *S*. *cerevisiae* genome (genome assembly EF4 downloaded from ENSEMBL) using Bowtie. Sequences were then assigned to genomic features using HTseq-count (mapping against the corresponding EF4 GTF file). Statistical significant enrichments of transcripts in the protein IPs relative to rRNA-depleted total RNA were determined using the Generalized Linear Model (GLM) functionality within edgeR to produce a comparison with a paired statistical design [54] and generated gene lists at a FDR<0.05. Datasets have been deposited at ArrayExpress, accession: E-MTAB-2892.

### Reverse Transcriptase PCR of total and Caf20-FLAG captured RNA

RNA isolation for qRT-PCR validation of Caf20-TAP IP targets (Fig 4B) was done as described above for RNA sequencing. To localise regions of Caf20-FLAG binding to specific transcripts (Fig 6F and S5 Fig) RNA isolation included the following modifications to the above FLAG affinity protocol. Cultures (2L) were grown to A_600_ = 0.6. For glucose starvation, cultures were split in two, harvested by centrifugation, and resuspendend in either prewarmed SC-URA (starvation) or SCD-URA (control) for 10 min. A formaldehyde cross-linking step was performed by rapidly chilling the culture with frozen media and adding formaldehyde to a final concentration of 1% (v/v) for 1 h with flasks kept on ice-water. Cross-linking was terminated with glycine to a final concentration of 0.1 M, and cultures pelleted and processed as described above. Following the final wash, the immunoprecipitated RNA was digested with 4U RNase III for 15 min, followed by proteinase K addition (50 μg) at 42°C for 30 min and heated to 65°C for 60 min to reverse crosslinks, as described previously [20]. The RNA was then processed to cDNA.

All isolated RNA was converted to cDNA using a Protoscript M-MuLV *Taq* RT-PCR kit (New England Biolabs, Ipswich, MA). For quantitative Reverse Transcriptase (qRT) PCR experiments (Figs 4B and 6F), Primer pairs were designed to *CAF20* and to a selection of Caf20p associated mRNAs (S2 Table). qRT-PCR was performed using the CFx Connect Real-Time system with iTaq Universal SYBR Green Supermix (BioRad Laboratories, Hercules, CA). Samples were run in triplicate and normalised to the input RNA. For semi-quantitative RT-PCR experiments (Fig 4E), PCR products were generated using Taq 2x Master Mix (New England Biolabs, Ipswich, MA) with a standard PCR program of 24 cycles and visualised on a 1% agarose gel containing SYBR Safe DNA Gel Stain (Life technologies, Paisley, UK).

For qPCR analysis of polysomal fractionated RNA. Gradient fractions were collected as described above into Trizol (Invitrogen). RNA was converted into cDNA and quantified as described above. 10 ng of a Luciferase mRNA control (Promega) was added to each fraction prior to RNA extraction to control for any influence variable concentrations of total RNA in each fraction may have during the RNA isolation and reverse transcription reactions. Data in Fig 7B are from technical duplicate samples and the proportion of each mRNA within a fraction was normalised to the Luciferase mRNA control. Once normalised, the total signal for a given mRNA across the gradient was determined and the amount of each mRNA is expressed as a percentage of the total signal across the gradient.

### RNA-sequencing and proteomics gene ontology analysis

Both protein and RNA gene lists were processed using the web based DAVID bioinformatics database [55] to assess the functional distribution of associated protein / RNA. Both protein and RNA datasets were FDR-corrected using standard protocols. A FDR cut off of 0.01 and Bonferroni correction were applied to the RNA datasets, whilst an FDR cut off of 0.05 were used with the protein datasets. Significantly enriched classifications were then visualised using standard Microsoft Excel tools.

### Structural and functional analyses of target RNAs

RNAs found to be enriched in the RIP-seq experiment were structurally and functionally characterised: gene location, orientation and structure, and mRNA PARS scores and folding were downloaded from http://genie.weizmann.ac.il/pubs/PARS10/pars10_catalogs.html [40] Poly A tail length and ribosome occupancy was obtained from [37], RNA half-life measurements were obtained from [38]. Continuous-data features were compared using Mann-Whitney test. Discrete features were tested for independence using Fisher’s exact test or χ^2^ test depending on the size of the contingency table. *P*-values for multiple comparisons were adjusted using Benjamini and Hochberg method [56].

### Motif discovery in UTR regions

5’ and 3’ UTR regions of mRNAs bound by Caf20p in 4E-DEP and-independent manners were searched for common sequence motifs using the MEME Suite [43]. Briefly, 1) each subset of sequences was used in discriminative motif discovery, where the motifs are specific to one subset and not specific to another subset of similar sequences; and, 2) the sensitivity and precision of the identified motifs were calculated by searching those motifs in the whole set of UTR sequences.

### Dual-luciferase assay

Strains GP7074–GP7082 were grown to A_600_ = 0.6 and protein samples prepared as above. Firefly and *Renilla* luciferase activity was quantified using a commercial dual luciferase assay (Dual-Glo Assay, Promega, UK). Briefly, 10 μg total protein extract was mixed with 250 μl of the Firefly luciferase substrate in a 1.5 ml cuvette and incubated for 20 min at room temperature. Firefly luciferase activity was measured on a GloMax 20/20 Luminometer (Promega). Stop-and-Glo reagent (250 μl) was then added and the *Renilla* luciferase activity was measured after a further 20 min incubation period at room temperature. *Renilla* activity was used to normalise firefly luciferase measurements.

## Supporting Information

S1 Table Yeast strains. (XLSX)

S2 Table Oligonucleotides. (XLSX)

S1 Fig Ribosome association of the 4E-BPs is disrupted by increased salt. Sucrose cushion centrifugation of cell extracts (input, I) into supernatant (S) and ribosome pellet (P) for wild-type cells (A and C) and corresponding mutants disrupted for eIF4E binding (B and D) in each 4E-BP. Western blots are shown for the indicated translation initiation factors, ribosomal subunit protein markers and Ahp1p a cytoplasmic marker protein (thioredoxin peroxidase). For each panel lanes 2–3 have standard buffer with 10 mM KCl, while 350 mM and 700 mM potassium acetate, respectively, was added to the buffer used for lanes 4–5 and 6–7.
(TIF)

S2 Fig Pairwise plots of Caf20 Rip Seq fold enrichments. Related to Fig 4C, the plots show log2 fold enrichments for individual mRNAs A) Caf20-TAP v Caf20-FLAG. B) Caf20-FLAG v Caf20^*m2*^-FLAG. Individual mRNAs are shown as circles colored based on their association <FDR 0.05 with each protein. Lines of best fit are shown in green, with 95% confidence intervals as broken lines. Insets show the relevant Venn sectors from Fig 4C.
(TIF)

S3 Fig Box and whisker plots comparing mRNA features of 4E-BP mRNA targets from RIp-Seq experiments. The analyses here expand upon those shown in Fig 6 presenting four additional data sets corresponding to TAP and FLAG RNA capture experiments described in the text. All comparisons used FDR<0.05 to select enriched mRNAs. Histograms above each plot show binned total data with the vertical dashed line indicating the median of the total. *P* values represent FDR (Mann-Whitney U tests corrected for multiple hypothesis testing). ‘Core’ gene sets were statistically tested versus the set of mRNAs not bound to TAP or FLAG tagged Caf20p. The TAP and FLAG full datasets were compared for significance testing with non-bound set for each experiment.
(TIF)

S4 Fig Median PARS scores across mRNA sets. Median PARS score for 4E-DEP (blue), 4E-IND (green) and non-Caf20p binding (grey) mRNAs. Each sequence section (UTR or coding sequence [CDS]) was described as a vector of length 100 containing the averaged PARS scores from 5’ (first value) to 3’ (100th value). As with a moving window average, the final score values depend on the neighbouring values; however, we made that influence decreased with the separation. Starting with the original PARS scores, if the section was longer than 100 nt, we averaged the PARS score of each pair of consecutive nucleotides. In this way, we shortened the scores vector in one value. We iterated until the length of the vector was equal to 100. If the section was shorter than 100 nt, we duplicated the length of the vector by assigning the value of the i^th^ position to the j^th^ and j+1^th^ (where j equals 2*i-1, and j+1 equals 2*i). We repeated the duplication process until the length of the vector was longer than 100; then, we proceeded as explained for longer sequences.
(TIF)

S5 Fig Caf20p interacts with ORFs and 4E-IND mRNA 3’UTRs. Fraction of each indicated mRNA isolated in complex with Caf20-FLAG (red) or Caf20^*m2*^-FLAG (blue bars) or an empty vector control (black) from *caf20*Δ cells following formaldehyde cross-linking and RNase III digestion. qRT-PCR detection with primer pairs hybridizing along each RNA as indicated by colour coding in each cartoon (right). Samples prepared from cells grown in SCD.
(TIF)

S1 Dataset Excel file with a series of sheets containing processed proteomics and sequencing data. Refer to the header sheet for details.
(XLSX)
