# Supplementary figures and images for: The 4E-BP Caf20p Mediates Both eIF4E-Dependent and Independent Repression of Translation
Source: PLoS Genet. 2015 May 14;11(5):e1005233. doi: 10.1371/journal.pgen.1005233 (PMC4431810; doi:10.1371/journal.pgen.1005233)

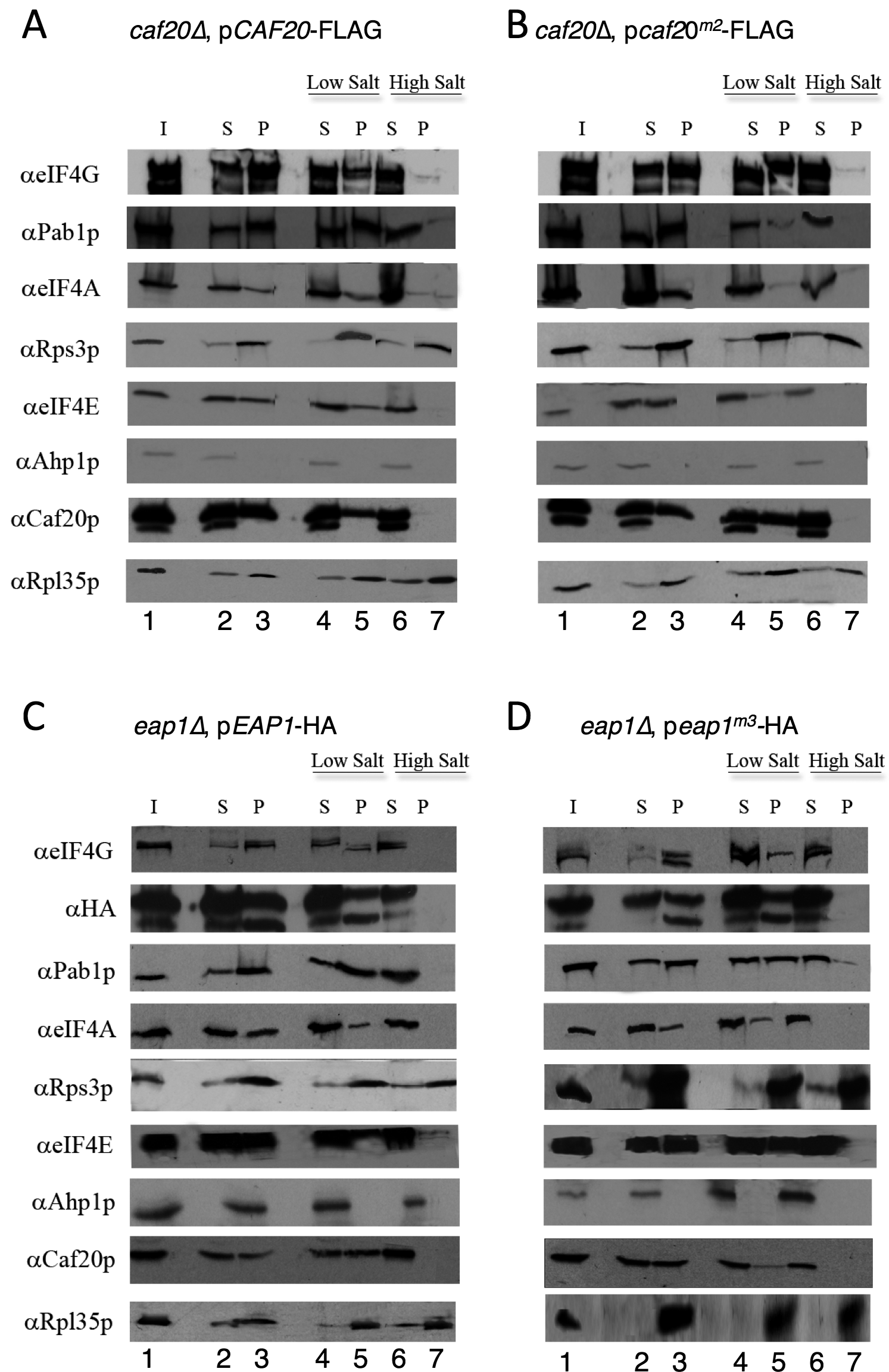

Supplement: S1 Fig — Sucrose cushion centrifugation of cell extracts (input, I) into supernatant (S) and ribosome pellet (P) for wild-type cells (A and C) and corresponding mutants disrupted for eIF4E binding (B and D) in each 4E-BP. Western blots are shown for the indicated translation initiation factors, ribosomal subunit protein markers and Ahp1p a cytoplasmic marker protein (thioredoxin peroxidase). For each panel lanes 2–3 have standard buffer with 10 mM KCl, while 350 mM and 700 mM potassium acetate, respectively, was added to the buffer used for lanes 4–5 and 6–7. (TIF) [file pgen.1005233.s003.tif]

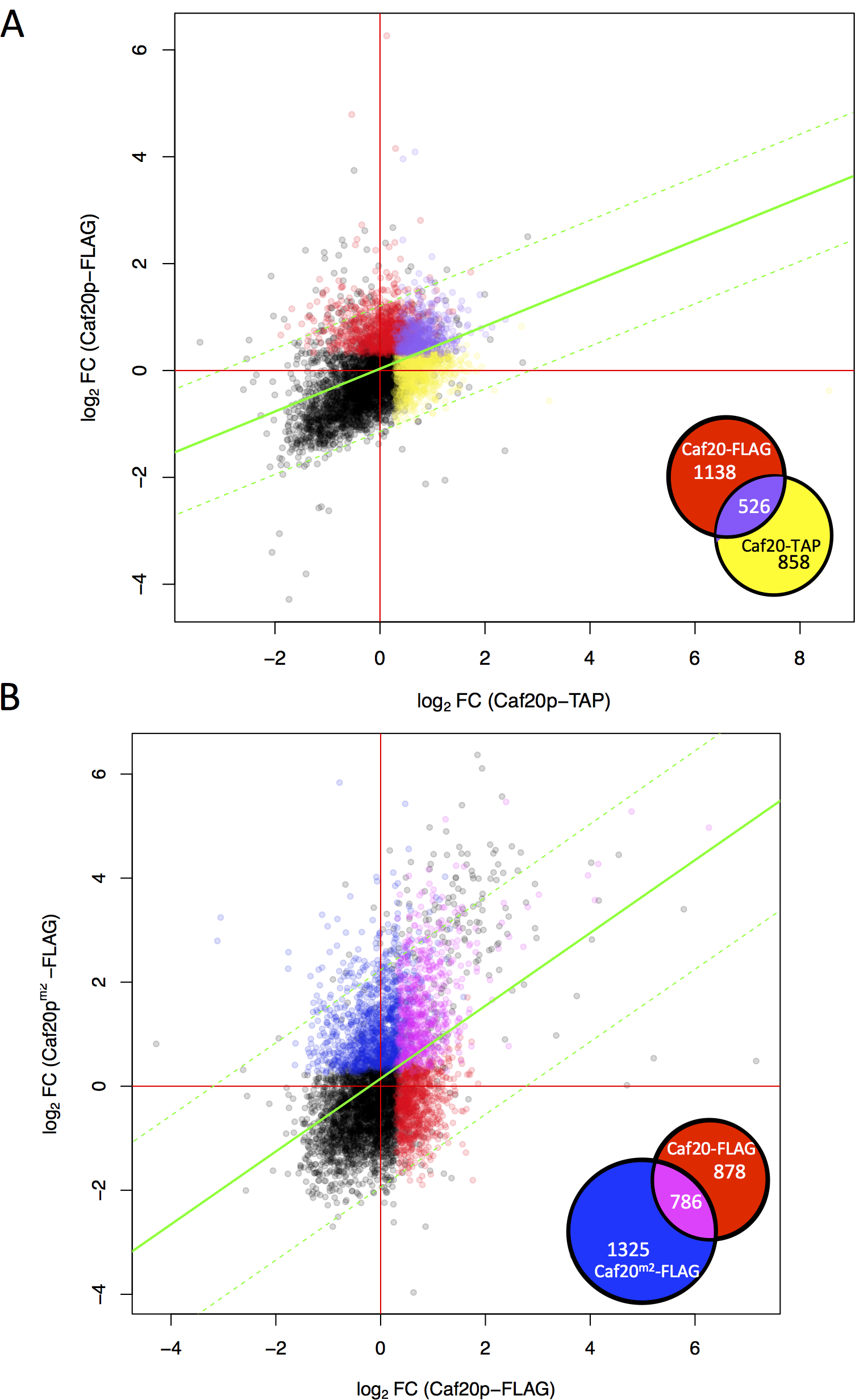

Supplement: S2 Fig — Related to Fig 4C, the plots show log2 fold enrichments for individual mRNAs A) Caf20-TAP v Caf20-FLAG. B) Caf20-FLAG v Caf20m2-FLAG. Individual mRNAs are shown as circles colored based on their association <FDR 0.05 with each protein. Lines of best fit are shown in green, with 95% confidence intervals as broken lines. Insets show the relevant Venn sectors from Fig 4C. (TIF) [file pgen.1005233.s004.tif]

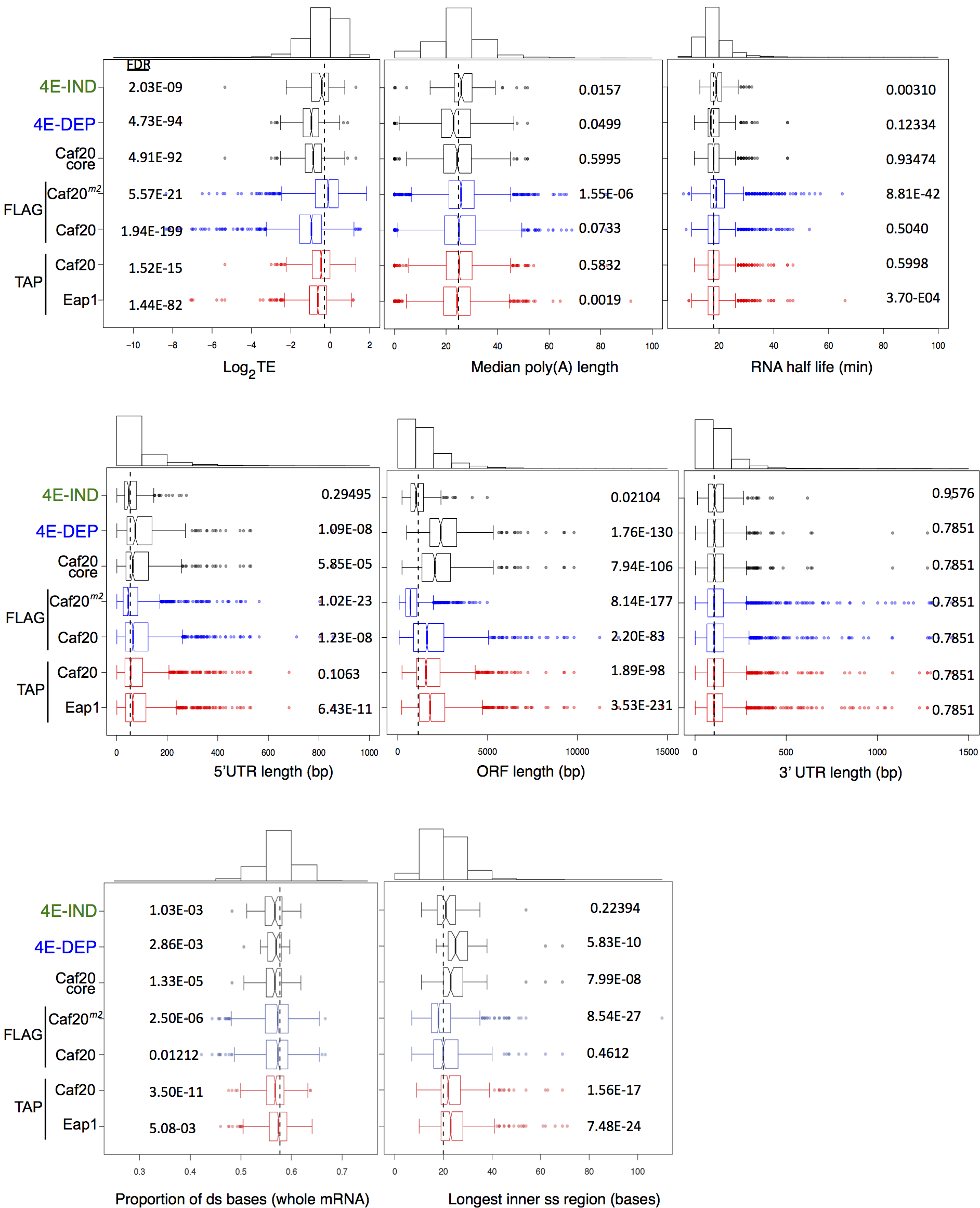

Supplement: S3 Fig — The analyses here expand upon those shown in Fig 6 presenting four additional data sets corresponding to TAP and FLAG RNA capture experiments described in the text. All comparisons used FDR<0.05 to select enriched mRNAs. Histograms above each plot show binned total data with the vertical dashed line indicating the median of the total. P values represent FDR (Mann-Whitney U tests corrected for multiple hypothesis testing). ‘Core’ gene sets were statistically tested versus the set of mRNAs not bound to TAP or FLAG tagged Caf20p. The TAP and FLAG full datasets were compared for significance testing with non-bound set for each experiment. (TIF) [file pgen.1005233.s005.tif]

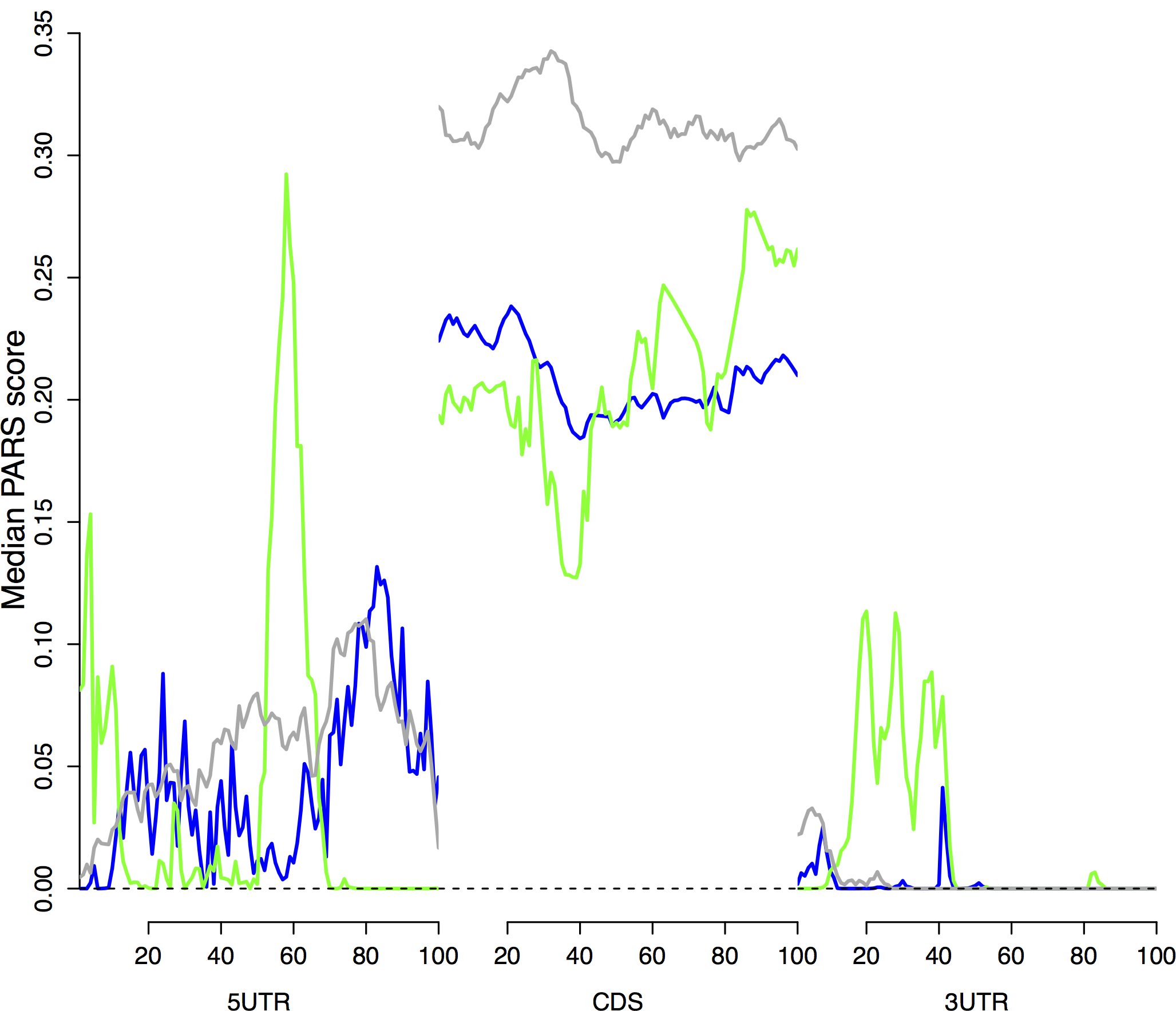

Supplement: S4 Fig — Median PARS score for 4E-DEP (blue), 4E-IND (green) and non-Caf20p binding (grey) mRNAs. Each sequence section (UTR or coding sequence [CDS]) was described as a vector of length 100 containing the averaged PARS scores from 5’ (first value) to 3’ (100th value). As with a moving window average, the final score values depend on the neighbouring values; however, we made that influence decreased with the separation. Starting with the original PARS scores, if the section was longer than 100 nt, we averaged the PARS score of each pair of consecutive nucleotides. In this way, we shortened the scores vector in one value. We iterated until the length of the vector was equal to 100. If the section was shorter than 100 nt, we duplicated the length of the vector by assigning the value of the ith position to the jth and j+1th (where j equals 2*i-1, and j+1 equals 2*i). We repeated the duplication process until the length of the vector was longer than 100; then, we proceeded as explained for longer sequences. (TIF) [file pgen.1005233.s006.tif]

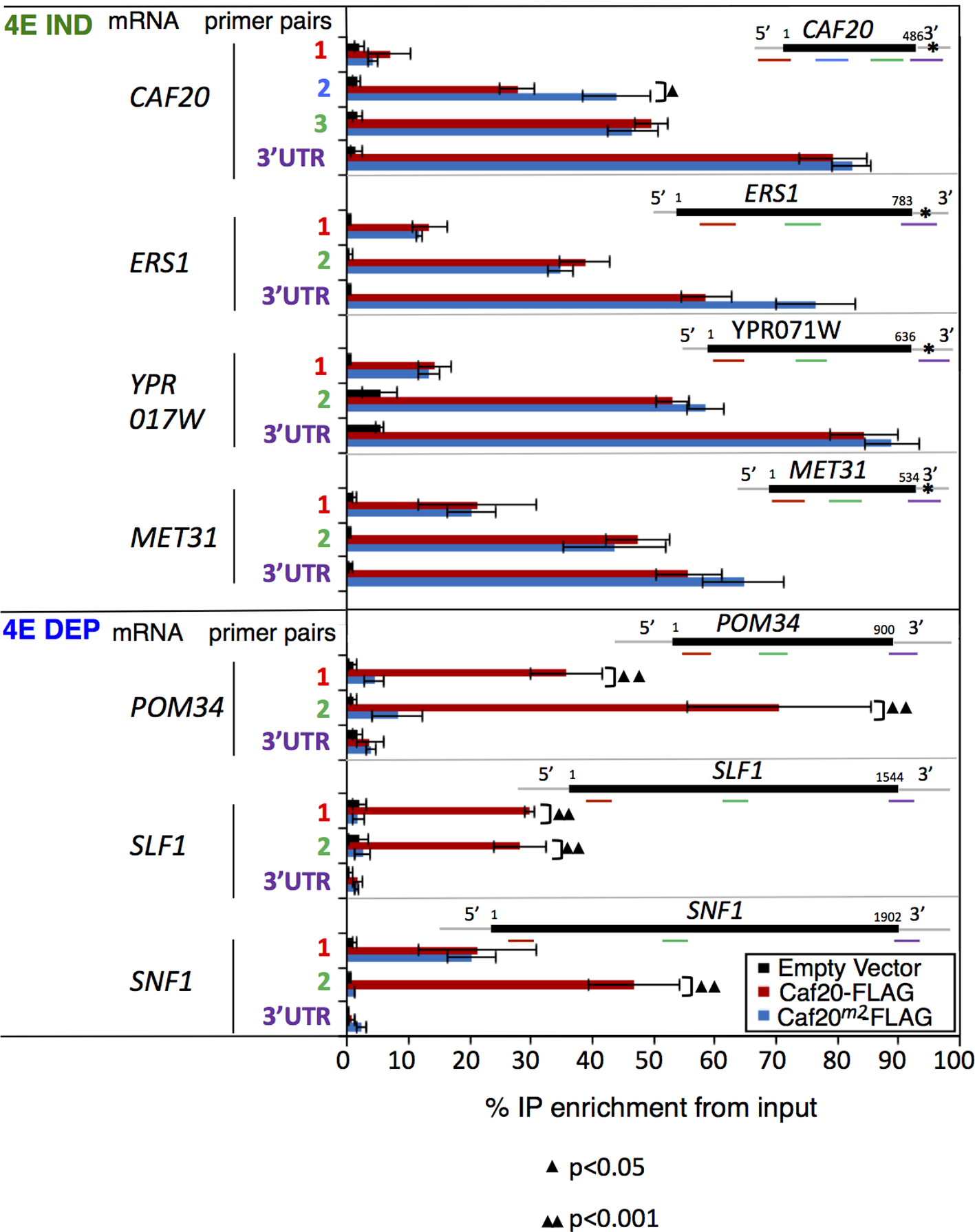

Supplement: S5 Fig — Fraction of each indicated mRNA isolated in complex with Caf20-FLAG (red) or Caf20m2-FLAG (blue bars) or an empty vector control (black) from caf20Δ cells following formaldehyde cross-linking and RNase III digestion. qRT-PCR detection with primer pairs hybridizing along each RNA as indicated by colour coding in each cartoon (right). Samples prepared from cells grown in SCD. (TIF) [file pgen.1005233.s007.tif]
